# Receptor clustering by a precise set of extracellular galectins initiates FGFR signaling

**DOI:** 10.1007/s00018-023-04768-x

**Published:** 2023-04-03

**Authors:** Dominika Zukowska, Aleksandra Gedaj, Natalia Porebska, Marta Pozniak, Mateusz Krzyscik, Aleksandra Czyrek, Daniel Krowarsch, Malgorzata Zakrzewska, Jacek Otlewski, Lukasz Opalinski

**Affiliations:** 1 Department of Protein Engineering, Faculty of Biotechnology, University of Wroclaw, Joliot-Curie 14a, 50-383 Wrocław, Poland; 2 Department of Protein Biotechnology, Faculty of Biotechnology, University of Wroclaw, Joliot-Curie 14a, 50-383 Wrocław, Poland

**Keywords:** FGFR, Galectins, N-Glycosylation, Receptor clustering, Multivalency, Signaling

## Abstract

FGF/FGFR signaling is critical for the development and homeostasis of the human body and imbalanced FGF/FGFR contributes to the progression of severe diseases, including cancers. FGFRs are N-glycosylated, but the role of these modifications is largely unknown. Galectins are extracellular carbohydrate-binding proteins implicated in a plethora of processes in heathy and malignant cells. Here, we identified a precise set of galectins (galectin-1, -3, -7, and -8) that directly interact with N-glycans of FGFRs. We demonstrated that galectins bind N-glycan chains of the membrane-proximal D3 domain of FGFR1 and trigger differential clustering of FGFR1, resulting in activation of the receptor and initiation of downstream signaling cascades. Using engineered galectins with controlled valency, we provide evidence that N-glycosylation-dependent clustering of FGFR1 constitutes a mechanism for FGFR1 stimulation by galectins. We revealed that the consequences of galectin/FGFR signaling for cell physiology are markedly different from the effects induced by canonical FGF/FGFR units, with galectin/FGFR signaling affecting cell viability and metabolic activity. Furthermore, we showed that galectins are capable of activating an FGFR pool inaccessible for FGF1, enhancing the amplitude of transduced signals. Summarizing, our data identify a novel mechanism of FGFR activation, in which the information stored in the N-glycans of FGFRs provides previously unanticipated information about FGFRs’ spatial distribution, which is differentially deciphered by distinct multivalent galectins, affecting signal transmission and cell fate.

**Supplementary Information:**

The online version contains supplementary material available at 10.1007/s00018-023-04768-x.

## Introduction

Fibroblast growth factor receptors (FGFRs) and their canonical ligands, and fibroblast growth factors (FGFs) form signaling platforms at the cell surface that regulate critical cellular processes, such as cell division, motility, differentiation, metabolism, and death [1]. The FGFR family comprises four receptors (FGFR1–FGFR4), the first three of which undergo alternative splicing, resulting in receptor isoforms with different ligand specificities and tissue expression profile [2, 3]. Pleiotropic and well-balanced FGF/FGFR activity is important for human development and homeostasis, while malfunctional FGFs/FGFRs are observed in numerous developmental diseases and cancers [4]. FGFR ligand selectivity, partial dimerization and activation of FGFRs in the absence of ligands, stability of FGF/FGFR pairs, co-receptor engagement, endocytosis, phosphatases, negative regulatory proteins, and negative feedback phosphorylation constitute the main known regulatory modules within FGF/FGFR that form a broad spectrum of diverse signaling outputs, ultimately shaping cell physiology [2, 5, 6].

FGFRs consist of three major parts: an N-terminal extracellular region that includes three Ig-like domains D1, D2 and D3, responsible for FGF binding, a single transmembrane helix that anchors FGFRs to the plasma membrane, and an intracellular split tyrosine kinase domain directly involved in phosphorylation events [3]. FGFRs are activated by at least 18 secreted FGFs with different specificities for distinct FGFR isoforms [1]. Binding of FGFs to the D2 and D3 domains of FGFRs induces receptor dimerization and conformational changes that trigger the sequential trans-phosphorylation of several tyrosine residues of the intracellular region of FGFRs [1, 7]. Activated FGFRs provide docking sites for adaptor proteins triggering signal propagation through several pathways: Ras/Raf-mitogen-activated protein kinase/extracellular signal regulated kinase kinase (MEK)–extracellular signal regulated kinase (ERK), phosphoinositide 3-kinase (PI3K)/protein kinase B (AKT)/mammalian target of rapamycin (mTOR), phospholipase Cγ (PLCγ), and signal transducer and activator of transcription (STAT) [1].

The extracellular regions of all four FGFRs are highly N-glycosylated at several positions, but the significance of FGFR N-glycosylation for FGF/FGFR signaling is still unclear and requires further investigation [8–11]. Duchesne et al*.* reported that N-glycosylation of FGFR1 modulates receptor’s interaction with FGF and heparan sulfate proteoglycans (HSPGs), co-receptors of FGFR [8]. N-glycosylation of FGFR2 was shown to regulate cellular trafficking and autoactivation of the receptor [12], and mutations in FGFR2 and FGFR3 that disrupt N-glycosylation were linked with craniosynostosis syndromes [11, 12].

**Fig. 1:**
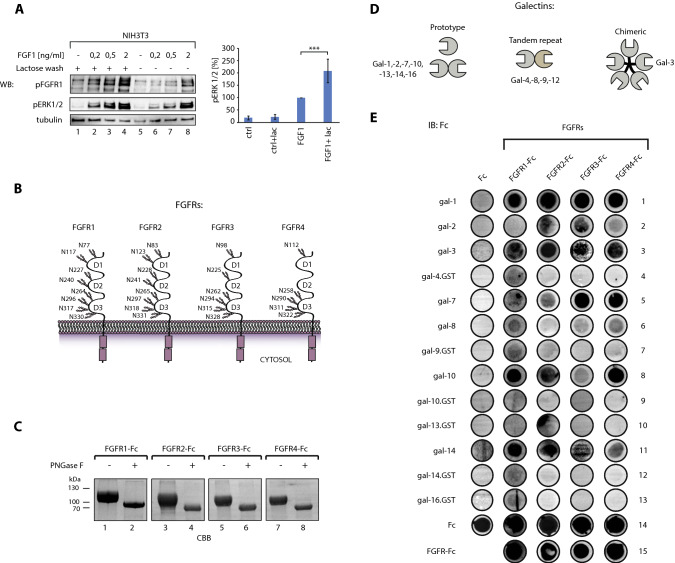
Galectins modulate FGF/FGFR signaling. **A** Effect of endogenous galectins on FGFR signaling. Serum-starved NIH3T3 cells were washed with 50 mM lactose prior the supplementation of cells with different concentrations of FGF1 (0.2–5 ng/mL). Cells were lysed and analyzed with WB using indicated antibodies. Tubulin served as a loading control (left panel). The densitometric analyses of the impact of lactose washes on the activation of FGFR-dependent signaling pathways by 5 ng/mL FGF1 (right panel). Average values from at least three independent experiments ± SEM are shown. Statistical analyses were performed with Student’s *t* test (*p < 0.05; **p < 0.005 and ***p < 0.001). **B** Scheme of FGFRs with N-glycosylation sites marked. **C** CBB stained gels of PNGase F treated recombinant FGFRs-Fc. **D** Schematic representation of the multivalent structures and classification of human galectins. Prototype galectins (galectin-1, -2, -7, -10, -13, -14, -16) contain a single carbohydrate recognition domain (CRD) that dimerizes. Tandem repeat galectins (galectin-4, -8, -9, -12) contain two distinct CRDs on a single polypeptide chain, while chimeric galectin-3 forms pentamers. **E** Galectin arrays to identify direct interactions between human galectins and FGFRs. Recombinant galectins were spotted onto the PVDF membrane and incubated with equimolar concentrations of the Fc fragment (control) or FGFRs-Fc. After extensive washing, Fc-bearing proteins interacting with individual galectins were detected with anti-Fc antibodies and chemiluminescence. Representative results from at least three independent experiments are shown

Galectins are a family of 12 soluble, secreted lectins in humans, implicated in a plethora of cellular processes, including signaling, cell proliferation, motility, endocytosis, autophagy and apoptosis, and in various diseases including cancers [13–15]. Based on their molecular architecture, galectins are divided into three groups: prototypic (galectin-1, -2, -7, -10, -13, -14, and -16), tandem-repeat (galectin-2, -8, -9, and -12) and chimeric (galectin-3) (Fig. 1C) [16]. Prototypic galectins consist of a single carbohydrate recognition domain (CRD) and can form dimers. Tandem-repeat galectins contain two different CRDs on a single polypeptide chain, while chimeric galectin-3 contains an N-terminal extension in addition to a single CRD, which facilitates high-order oligomerization (Fig. 1C) [17]. Galectins are able to bind the N-linked sugar chains of glycoproteins containing β-galactosides and affect glycoprotein function, co-receptor access and cellular transport [17, 18].

Indirect involvement of galectins in the regulation of FGFR-dependent signaling was initially demonstrated by Ming et al., showing that galectin-3 binds N-glycosylated Klotho-β and thereby modulates Klotho-β accessibility for the FGF21/FGFR1 signaling complex [19]. Galectin-3 was detected in complex with N-glycosylated integrin αvβ3, regulating FGF2-dependent angiogenesis [20]. Recently, we have provided the first evidence of a direct N-glycosylation-dependent interaction between galectin-1 and -3 and FGFR1, leading to FGFR1 activation, stimulation of cell division, and avoidance of apoptosis [21]. In addition to galectin-1 and -3, galectin-7 and -8 were also identified as putative FGFR1-binding proteins in two separate high-throughput screens, but their involvement in FGFR1-dependent signaling has never been investigated [21, 22].

Here, we have performed the first comprehensive and detailed analysis of the interplay between all human galectins and FGFRs. We provide robust evidence for the presence of a novel regulatory module in FGF/FGFR signaling, where N-glycosylation of the receptor provides an additional layer of information that is differentially decoded by different members of the galectin family to fine-tune FGF/FGFR signaling and determine cell performance.

## Results

### Screening reveals four galectins as binding partners for FGFRs

Since the extracellular regions of FGFRs are N-glycosylated at several positions, FGFRs’ signaling can be directly controlled by secreted galectins. To study whether endogenous galectins can modulate FGFR signaling, we washed serum-starved NIH3T3 model fibroblasts producing predominantly FGFR1 with lactose to deprive cells of cell surface-associated galectins and assessed FGFR1 activation by FGF1. Treatment of cells with lactose significantly increased the activation of FGFR1 and ERK1/2 by FGF1, indicating a role of galectins in modulating FGFR1 signaling (Fig. 1A). Treatment of NIH3T3 cells with mannose, a galectin non-binding sugar, had no effect on FGF1 signaling (Fig. S1).

The FGFR family in human includes four receptors: FGFR1, FGFR2, FGFR3, and FGFR4, which contain eight, eight, six, and five putative N-glycosylation sites (Fig. 1B) [8, 9, 13, 23, 24]. We produced extracellular regions of FGFR1–FGFR4 as fusions to the Fc fragment of human IgG1 in CHO cells and confirmed their N-glycosylation with PNGase F treatment (Fig. 1C).

The human galectin family consists of 12 proteins that differ in their oligomeric state and carbohydrate specificity (Fig. 1D). To screen for galectins capable of binding FGFRs, we produced all human recombinant galectins either as His-Tag or glutathione *S*-transferase fusions. SDS-PAGE and western blotting analyses confirmed the identity and revealed the high purity of recombinant galectins (Fig. S2A–B). Pull-down experiments with lactose–agarose beads revealed that all obtained galectins were functional (Fig. S2C).

Next, we developed a robust protocol to identify human galectins that interact with FGFRs. To this end, recombinant galectins or positive controls (FGFRs-Fc and Fc), were immobilized on PVDF membrane to form a galectin dot blot array. After blocking, the galectin arrays were incubated with equimolar concentrations of FGFRs-Fc or Fc (negative control). After extensive washing, complexes between individual galectin and FGFRs were detected using HRP-conjugated anti-Fc antibody and chemiluminescence (Fig. S3). The anti-Fc antibody detected spots with positive controls: FGFRs-Fc and Fc, confirming the feasibility of the approach taken (Fig. 1E, rows 14 and 15). We observed an enhanced signal for galectin-1, -3, -4, -7, -8, -9, -10, -14, and -16 for FGFR1-Fc in relation to the Fc control, with the strongest signals detected for galectin-1, -3, -10, and -14 (Fig. 1E, rows 1, 3, 8, and 11). We detected positive signals corresponding to galectin-1, -2, -3, -7, -10, and -14 for FGFR2-Fc (Fig. 1E, rows 1, 2, 3, 5, 8, and 11), and galectin-1, -2, -3, -7, -8, -10, and -14 for FGFR3-Fc and FGFR4-Fc (Fig. 1E, rows 1, 2, 3, 5, 6, 8, and 11). These data indicate that different galectins directly interact with glycosylated FGFRs.

**Fig. 2:**
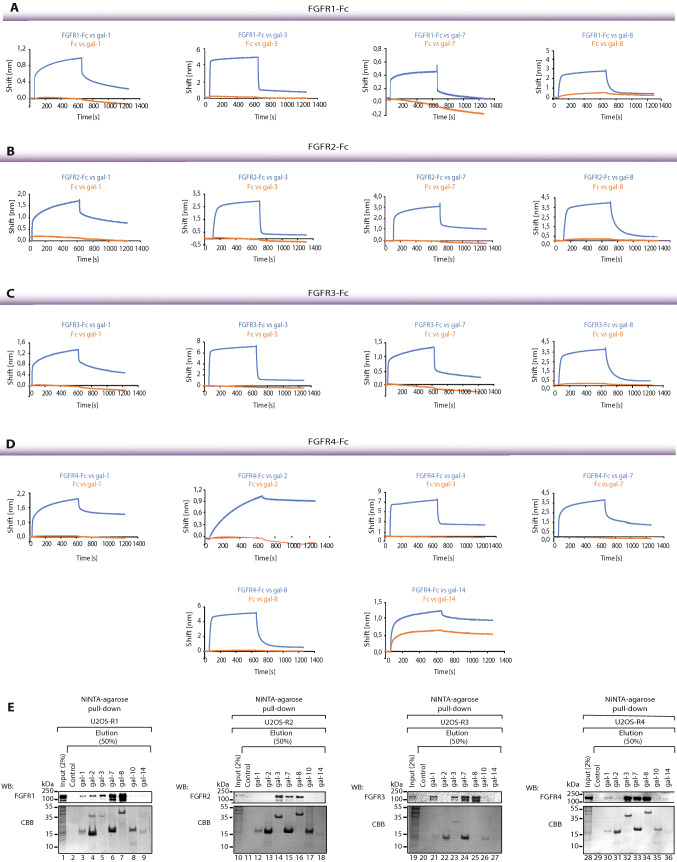
Galectins directly interact with FGFRs. BLI analyses of interactions between FGFRs and galectins. FGFR1-Fc (**A**), FGFR2-Fc (**B**), FGFR3-Fc (**C**) and FGFR4-Fc (**D**) were immobilized on Protein-A biosensors (blue lines) in a pairwise fashion with equimolar concentrations of Fc (control; red lines) and incubated with recombinant galectins to record association and dissociation phases. Representative results from at least three independent experiments are shown. **E** Pull-down experiments of recombinant galectins with a cellular pool of FGFRs. U2OS-R1/R2/R3/R4 cells were lysed and the lysates were incubated with recombinant galectins bound to NiNTa resin. After washing, galectin-bound proteins were analyzed with WB using antibodies against FGFRs. CBB staining depicts mainly recombinant galectins in eluted fractions. Representative results from at least three independent experiments are shown

To confirm these findings, we used biolayer interferometry (BLI). FGFRs-Fc and the Fc fragment were immobilized on Protein-A BLI biosensors in a pairwise manner, incubated with recombinant galectins, and the association and dissociation stages of the interaction between the proteins tested were monitored. The positive BLI signals were obtained for galectin-1, -3, -7, and -8, which bound all FGFRs (Fig. 2A–D). Galectins differed in their FGFR-binding profiles (slow association and slow dissociation phases for galectin-1, fast association, and fast dissociation phases for galectin-3, -7, and -8) (Fig. 2A–D). Interestingly, BLI experiments revealed an interaction between galectin-2 and -14, and FGFR4-Fc that was insignificant for other FGFRs (Fig. 2D). Since galectin-14 also interacted with control Fc in BLI (although a much lower signal was detected than for FGFR4-Fc), we decided to omit this galectin in subsequent studies. For the other galectins that were identified with the galectin array, we detected either no signal or a very weak signal in BLI measurements, or there was non-specific binding of galectins to the sensor/the Fc fragment. Importantly, galectins-1, -2, -3, -7, and -8 displayed no binding to the Fc fragment, confirming the specificity of the interaction between galectins and FGFRs (Fig. 2A–D).

Since the N-glycosylation pattern of mouse and human cells can differ [25, 26], we studied whether galectin-1, -2, -3, -7, and -8 interact with full-length FGFRs possessing human N-glycosylation type. We applied pull-down experiments with U2OS cells stably transfected with FGFR1 (USOS-R1), FGFR2 (U2OS-R2), FGFR3 (U2OS-R3), and FGFR4 (U2OS-R4). Recombinant galectins were bound in equimolar concentrations to Ni–NTA beads and incubated with lysates prepared from U2OS-R1, U2OS-R2, U2OS-R3, and U20S-R4 cells. Co-purification of FGFRs with individual galectins was assessed with western blotting. As shown in Fig. 2E, the most effective co-purification of all four FGFRs was observed with galectin-3, -7, and -8 (lanes 5–7, 14–16, 23–25, 32–34). Galectin-1 co-purified with FGFR1, FGFR3, and FGFR4, albeit with lower efficiency than galectin-3, -7, and -8 (Fig. 2E, lanes 3, 21, and 30), while galectin-2 weakly co-purified only with FGFR1 (Fig. 2E, lane 4). Since N-glycans are heterogeneous molecules that are synthesized with different monosaccharides at their termini, and that the process of N-glycan synthesis is cell type- and tissue type-dependent [27–30], we studied the interaction between galectins-1, -3, -7, and -8 and FGFR1 expressed by a panel of human cell lines: human epithelial breast cancer cells (JIMT-1), osteosarcoma cells (G292) and lung small cell carcinoma cells (DMS114). As shown in Fig. S4, all of galectins tested interact with differentially N-glycosylated FGFR1 of human origin. Additionally, using BLI, we demonstrated that galectin-1, -3, -7, and -8 directly interact with human recombinant FGFR1 (Fig. S5).

These data indicate that endogenous galectins modulate FGFR signaling. Furthermore, our data suggest that galectins directly interact with all four FGFRs, and galectin-1, -3, -7, and -8 emerge as the most effective binders of FGFRs among human galectins.

### Galectins interact with FGFRs with sub-micromolar affinity via N-linked glycans

**Fig. 3:**
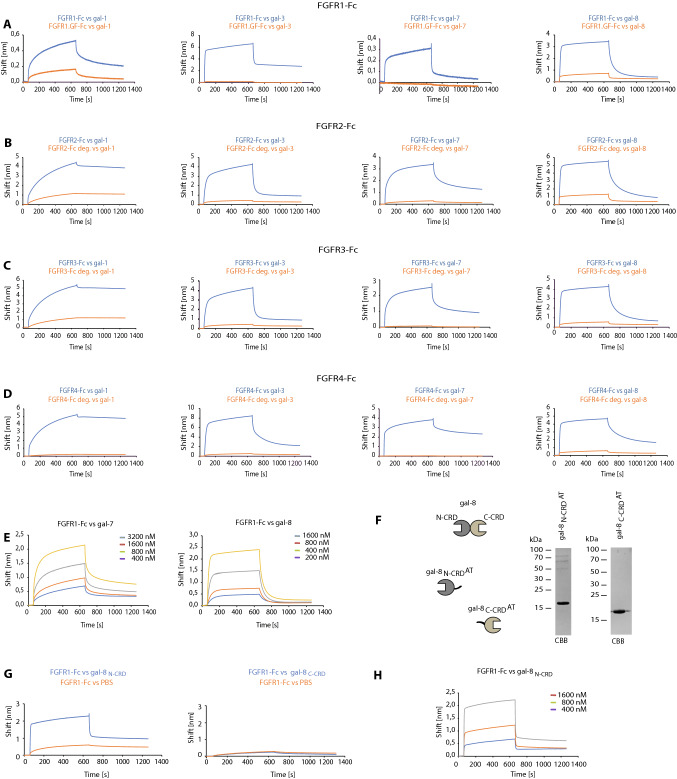
Galectins recognize the N-glycans of FGFRs. **A**–**D** BLI analyses of the interaction between N-glycosylated and de-glycosylated FGFRs, FGFs, and galectins. FGFR1-Fc (**A**), FGFR2-Fc (**B**), FGFR3-Fc (**C**), and FGFR4-Fc (**D**) were immobilized on Protein-A biosensors (blue lines) in a pairwise fashion with equimolar concentrations of the N-glycosylation-deficient mutant of FGFR1 (FGFR1.GF-Fc) or FGFR2-Fc, FGFR3-Fc, and FGFR4-Fc treated with PNGase F (red lines) and incubated with recombinant galectins to record the association and dissociation phases. Representative results from at least three independent experiments are shown. **E** BLI measurements of the kinetics of interaction between FGFR1-Fc, galectin-7 and -8. Kinetics values are provided in Table 1. **F** CBB stained gels of recombinant single CRD variants of galectin-8. **G** BLI binding curves of FGFR1-Fc with gal8_N-CRD_ and gal8_C-CRD_ variants. **H** BLI measurements of the kinetics of interaction between FGFR1-Fc and gal8_N-CRD_; binding parameters are provided in Table 1

We tested whether the direct interaction between the identified set of galectins and FGFRs occurs via FGFR-linked N-glycans. To this end, we prepared a recombinant, glycosylation-free mutant of the extracellular region of FGFR1 fused to Fc (FGFR1.GF-Fc) and confirmed its proper folding by FGF2-binding assay using BLI (Fig. S6A and B). Equal amounts of FGFR1-Fc and FGFR1.GF-Fc were immobilized on BLI sensors and incubated with recombinant galectins. As shown in Fig. 3A, virtually no interaction between FGFR1.GF-Fc and galectin-1, -3, -7, and -8 was detected. For other FGFRs, we enzymatically removed the N-linked sugar chains using PNGase F, yielding de-glycosylated receptors: FGFR2.deg-Fc, FGFR3.deg-Fc, and FGFR4.deg-Fc (Fig. 1C). FGFR2-Fc/FGFR2.deg-Fc, FGFR3-Fc/FGFR3.deg-Fc, and FGFR4-Fc/FGFR4.deg-Fc pairs were then compared for binding of selected galectins using BLI. The interaction of FGFR2-Fc, FGFR3-Fc, and FGFR4-Fc with all tested galectins was almost completely abolished after enzymatic removal of N-linked sugars from the receptors (Fig. 3B–D). We also performed BLI measurements of the interaction between galectin-1, -3, -7, and -8 and FGFR1-Fc in the presence of lactose or mannose. As shown in Fig. S7, mannose was unable to block the binding of the tested galectins to FGFR1-Fc, whereas lactose fully inhibited the interaction of galectin-1, -3, -7, and -8 with FGFR1-Fc.

The kinetic parameters of the interaction between galectin-1 and -3, and FGFR1 were reported by us previously [21]. We measured now the affinity of galectin-7 and -8 for FGFR1-Fc and, additionally, all galectins tested for FGFR2-Fc, FGFR3-Fc, and FGFR4-Fc. As shown in Fig. 3E and Table 1, the strongest, sub-micromolar affinity was measured for galectin-8 and FGFR1. Other galectins bind FGFRs with micromolar affinity, but differ in *k*_on_/*k*_off_ values, indicating different half-lives of the individual galectin–FGFR complexes (Table 1).

**Table 1:** Kinetic parameters of the interaction between the studied galectins and FGFRs

	*K*_D1_ [M]	*K*_D2_ [M]	*K*_on1_ [M^−1^ s^−1^]	*K*_on2_ [M^−1^ s^−1^]	*K*_Off1_ [s^−1^]	*K*_Off2_ [s^−1^]
FGFR1-Fc						
gal-1 [21]	1.69E−06	6.03E−06	1.07E+03	1.23E+03	1.81E−03	9.20E−02
gal-3 [21]	2.60E−06	1.34E−08	3.68E+04	6.12E+04	9.66E−03	8.21E−02
gal-7	9.65E−06	3.31E−06	4.95E+03	2.20E+03	5.47E−02	6.72E−04
gal-8	9.50E−07	1.94E−07	2.52E+04	8.82E+03	2.25E−02	7.66E−04
gal-8_N-CRD_	2.60E−06	1.70E−08	1.65E+05	4.29E+05	1.64E−01	1.94E−04
FGFR2-Fc						
gal-1	6.77E−07	1.11E−07	2.77E+05	9.87E+03	1.68E−01	7.87E−04
gal-3	2.22E−05	6.51E−05	1.45E+03	2.21E+03	1.03E−03	0.40E−01
gal-7	7.83E−06	1.40E−06	1.66E+04	3.48E+03	1.20E−01	2.12E−03
gal-8	3.80E−06	1.41E−07	1.32E+04	3.51E+04	3.12E−02	4.93E−03
FGFR3-Fc						
gal-1	1.17E−07	1.98E−07	3.84E+03	8.29E+04	2.66E−04	2.05E−02
gal-3	1.165E−04	1.18E−04	4.13E+03	1.87E+05	2.05E−02	2.14E−02
gal-7	2.55E−06	4.22E−07	4.24E+04	5.65E+05	1.31E−01	5.23E−04
gal-8	1.74E−07	6.30E−08	1.67E+05	1.45E+04	2.89E−02	8.55E−04
FGFR4-Fc						
gal-1	1.77E−08	1.51E−07	1.02E+04	6.80E+05	1.88E−04	9.79E−02
gal-3	2.67E−05	4.27E−06	3.83E+02	1.55E+04	3.42E−03	2.90E−02
gal-7	2.73E−05	3.21E−08	6.08E+03	2.90E+05	4.19E−03	1.05E−02
gal-8	2.43E−08	2.49E−07	3.87E+04	6.51E+04	9.29E−04	1.61E−02

Galectin-8 is a tandem repeat galectin composed of two CRDs with distinct carbohydrate specificities (Fig. 3F) [31]. We produced separate CRDs of galectin-8 fused to the AviTag (gal-8_N_-_CRD_ and gal-8_C-CRD_) (Fig. 3F) and demonstrated that only gal-8_N_-_CRD_ interacts with FGFR1-Fc (Fig. 3G). Interestingly, the binding profiles of gal-8_N-CRD_ to FGFR1-Fc were virtually identical to the curves obtained for the full-length galectin-8, indicating that N-CRD is exclusively responsible for the recognition of FGFR1-Fc (Fig. 3H).

All these data demonstrate that four galectins interact directly and with different kinetics with N-glycans attached to FGFRs. Based on the outcome of the experiments described above, we decided to focus in further studies on FGFR1 as exemplary FGFR and galectins-1, -3, -7, and -8.

### Galectins recognize N-linked glycans on the membrane-proximal D3 domain of FGFR1, inducing receptor crosslinking

**Fig. 4:**
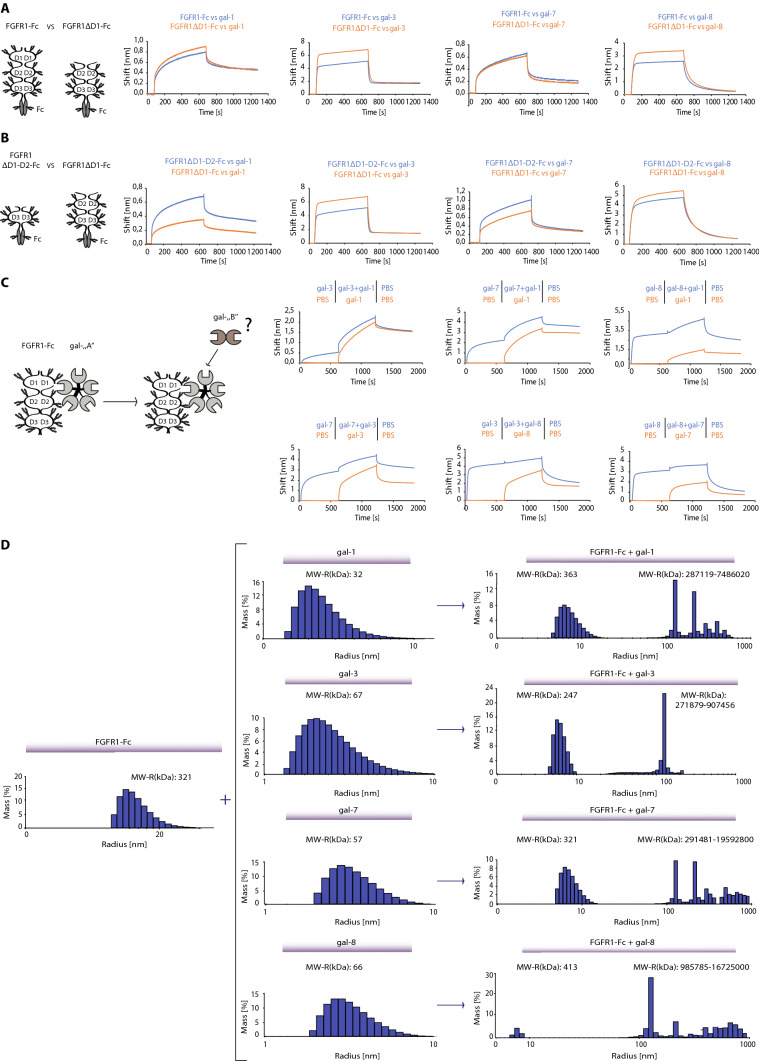
Galectins bind the N-glycans of the D3 domain of FGFR1 and induce differential clustering of the receptor. BLI analyses of the interaction between the full length FGFR1-Fc or a truncated variant of the receptor lacking the D1 domain (FGFR1ΔD1-Fc) (**A**); or FGFR1ΔD1-Fc and receptor truncation lacking the D1 and D2 (FGFR1ΔD1-D2-Fc) (**B**) and selected galectins. Equimolar concentrations of the proteins tested were bound to Protein-A sensors, incubated with recombinant galectins and association and dissociation were recorded with BLI. Schematic structures of receptor variants are shown (left panel). Representative results from at least three independent experiments are shown. **C** BLI epitope binning experiments with FGFR1-Fc and galectins (experimental scheme shown in the left panel). FGFR1-Fc was immobilized on Protein A sensors and incubated with saturating concentrations of first galectin or buffer (control). The sensors were then moved to a solution containing the second galectin and BLI curves were compared between the test set-up and control. Representative results from at least three independent experiments are shown. **D** Galectins induce clustering of FGFR1. DLS signals of recombinant FGFR1-Fc (left panel), galectins (middle panels) and mixtures of these proteins (right panels) are shown. DLS-estimated MW of proteins are shown. High molecular weight complexes are seen upon incubation of FGFR1-Fc and galectins, which are not detected in single protein samples

To locate the binding sites for individual galectins on FGFR1, we used FGFR1-Fc containing eight N-glycosylation sites, FGFR1.D2-D3-Fc truncated variant lacking the N-terminal D1 domain, which contains two N-glycosylation sites, and FGFR1.D3-Fc with the remaining four N-glycosylation sites (Fig. 4A and B). Truncated variants of FGFR1 were produced in CHO cells and their N-glycosylation was confirmed with PNGase F treatment (Fig. S8A and B). To assess whether the N-linked glycans attached to the D1 domain of FGFR1 provide binding sites for galectins, we used BLI with immobilized FGFR1-Fc and FGFR1.D2-D3-Fc and searched for a significant reduction in BLI signals for FGFR1.D2-D3-Fc and individual galectin in relation to FGFR1-Fc. As shown in Fig. 4A, no decrease in BLI signal was observed for any of the galectins tested after removal of the D1 domain of the receptor. The BLI curves of galectins with FGFR1.D2-D3-Fc (with two and four N-glycosylation sites in the D2 and D3 domains, respectively) and FGFR1.D3-Fc (containing membrane-proximal four N-glycosylation sites) were then compared. BLI experiments revealed highly similar interaction between all tested galectins and FGFR1.D2-D3-Fc, and FGFR1.D3-Fc, indicating that the binding sites for galectin-1, -3, -7, and -8 are localized within the D3 domain of FGFR1 (Fig. 4B).

Next, we applied an epitope binding approach with BLI to test if individual galectins compete for binding sites on the D3 domain of FGFR1. FGFR1-Fc was immobilized on BLI sensors, and the sensors were incubated with either the first galectin (sample sensor) or buffer (reference sensor). The sensors were then transferred to a solution containing a mixture of first and second galectin (sample sensors; to avoid the mixed effect of dissociation of the first galectin and association of the second one) or second galectin only (control sensor). The effect of binding of the first galectin to FGFR1-Fc on the interaction of the second galectin with the receptor was assessed by comparing the binding profile of the second galectin to FGFR1-Fc relatively to the FGFR1-Fc—first galectin complex (Fig. 4C). Galectin-1 only partially competes for FGFR1 binding with galectin-3, -7, and -8, as the association of galectin-1 with FGFR1-Fc was only mildly altered in the presence of the tested galectins. In contrast, galectin-3, -7, and -8 compete with each other for FGFR1-Fc, as binding of any of these galectins significantly blocked the binding of the other (Fig. 4C).

We applied dynamic light scattering (DLS) to probe whether the studied galectins induce FGFR1 crosslinking. DLS measurements revealed that all of the tested galectins induce the assembly of a wide range of very high molecular weight complexes consisting of FGFR1-Fc and galectins (Fig. 4D). Surprisingly, we detected FGFR1-Fc clustering with galectin-8, which binds FGFR1 predominantly with N-CRD, indicating that C-CRD may still partially contribute to FGFR1 clustering.

These data indicate that all four selected galectins bind at least one of the four N-linked sugars within the D3 domain of FGFR1. Furthermore, our data suggest that galectins interact with partially distinct N-linked sugars on the D3 of the FGFR1, leading to differential receptor clustering.

### Receptor crosslinking constitutes a mechanism for galectin-mediated activation of FGFR1 and receptor-downstream signaling

**Fig. 5:**
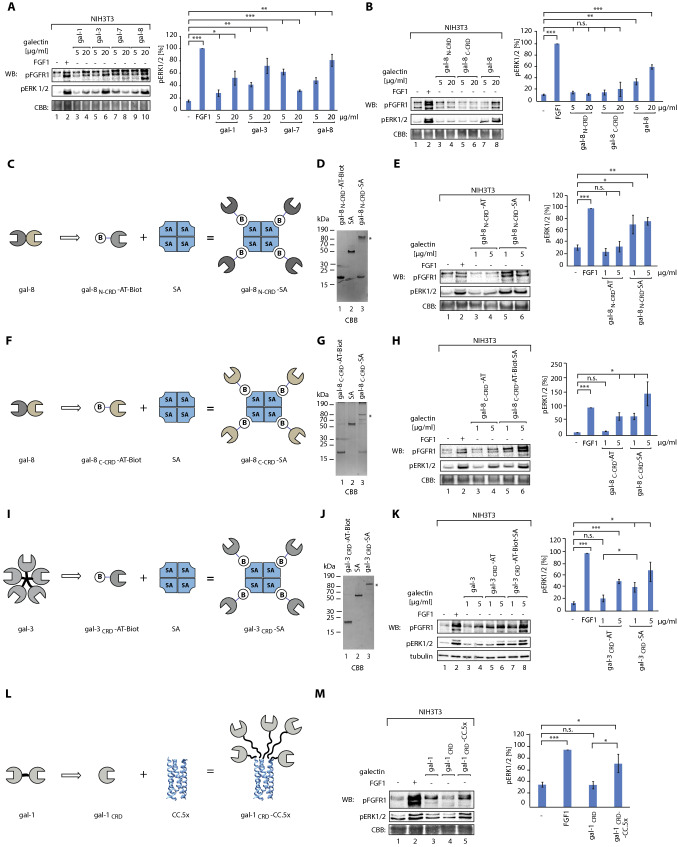
Multivalency of galectins is critical for activation of FGFR1 and receptor-downstream signaling pathways. **A** and **B** Effects of wild type galectins and individual CRDs of galectin-8 on FGFR1 signaling. Serum-starved NIH3T3 cells were treated with FGF1 (100 ng/mL, control) or recombinant galectins. Cells were lysed and analyzed with WB using the indicated antibodies. CBB served as a loading control (left panel). Densitometric analyses of the effect of galectins on ERK1/2 activation (right panel). Mean values from at least three independent experiments ± SEM are shown. **C** Scheme of the strategy applied to generate monovalent and tetravalent gal-8_N-CRD_. AviTagged-gal-8_N-CRD_ was site-specifically biotinylated with BirA and incubated with tetrameric streptavidin (SA), leading to self-assembly of gal-8_N-CRD_-SA tetramers, which were purified from the individual components by gel filtration. **D** SDS-PAGE analyses of the assembly of tetravalent gal-8_N-CRD_-SA. Mixing biotinylated gal-8_N-CRD_ (lane 1) with SA (lane 2) results in the formation of a highly stable (non-denatured by SDS) HMW complex (*), which corresponds to tetravalent gal-8_N-CRD_-SA (lane 3). **E** Effects of monovalent gal-8_N-CRD_ and tetravalent gal-8_N-CRD_-SA on FGFR1 signaling. Serum-starved NIH3T3 cells were treated with FGF1 (100 ng/mL, control) or recombinant galectins, cells were lysed and analyzed with WB using the indicated antibodies (left panel). CBB served as a loading control. Densitometric analyses of the effect of gal-8_N-CRD_ variants with different valency on the level of pERK1/2 (right panel). Mean values from at least three independent experiments ± SEM are shown. **F** Scheme of strategy applied to generate monovalent and engineered tetravalent gal-8_C_-_CRD_ (as in panel **C**). **G** SDS-PAGE analyses of the assembly of tetravalent gal-8_C-CRD_-SA. Mixing biotinylated gal-8_C-CRD_ (lane 1) with SA (lane 2) results in the formation of a highly stable (non-denatured by SDS) HMW complex (*), which corresponds to tetravalent gal-8_C-CRD_-SA (lane 3). **H** Effects of monovalent gal-8_C-CRD_ and tetravalent gal-8_C-CRD_-SA on FGFR1 signaling. Serum-starved NIH3T3 cells were treated with FGF1 (100 ng/mL, control) or recombinant galectins, cells were lysed and analyzed with WB using the indicated antibodies (left panel). CBB served as a loading control. Densitometric analyses of the effect of gal-8_C-CRD_ variants of different valency on the level of pERK1/2 (right panel). Mean values from at least three independent experiments ± SEM are shown. **I** Strategy used for to develop monovalent and tetravalent gal-3_CRD_ (as in panel C). **J** SDS-PAGE analyses of the assembly of tetravalent gal-3_CRD_-SA. Mixing biotinylated gal-3_CRD_ (lane 1) with SA (lane 2) results in the formation of a highly stable (non-denatured by SDS) HMW complex (*), which corresponds to tetravalent gal-3_CRD_-SA (lane 3). **K** Effects of monovalent gal-3_CRD_ and tetravalent gal-3_CRD_-SA on FGFR1 signaling. Serum-starved NIH3T3 cells were treated with FGF1 (100 ng/mL, control), or recombinant galectins, cells were lysed and analyzed with WB using the indicated antibodies (left panel). Tubulin served as a loading control. Densitometric analyses of the effect of gal-3_CRD_ variants of different valency on the level of pERK1/2 (right panel). Mean values from at least three independent experiments ± SEM are shown. **L** The strategy used to generate monovalent gal-1_CRD_ and multivalent gal-1_CRD_.CC.5x. N-terminus responsible for galectin-1 oligomerization was deleted, yielding monovalent gal-1_CRD._ Gal-1_CRD_ was fused with a pentamerizing coiled-coil sequence (CC.5x), resulting in a self-assembling multivalent gal-1_CRD_.CC.5x. **M** Effects of monovalent gal-1_CRD_ and multivalent gal-1_CRD_.CC.5x on FGFR1 signaling. Serum-starved NIH3T3 cells were treated with FGF1 (100 ng/mL, control) or recombinant galectins, cells were lysed and analyzed with WB using the indicated antibodies (left panel). CBB served as a loading control. Densitometric analyses of the effect of galectin-1 variants of different valency on the level of pERK1/2 (right panel). Mean values from at least three independent experiments ± SEM are shown. Statistical analyses were performed with Student’s *t* test (*p < 0.05; **p < 0.005 and ***p < 0.001)

We studied the effect of galectins binding to N-linked sugars of FGFR1 on receptor activation and induction of FGFR1-dependent signaling pathways using model NIH3T3 fibroblasts and western blotting. Treatment of serum-starved cells with galectin-1, -3, and -8 resulted in a dose-dependent increase in phosphorylated FGFR1 (pFGFR1) and downstream kinases ERK1/2 (pERK1/2) (Fig. 5A). We also observed a significant increase in pFGFR1 and pERK1/2 signals upon supplementation of cells with galectin-7, but for this galectin, we detected higher activation of signaling at lower concentration (Fig. 5A, lanes 7 and 8). The galectins tested were unable to activate FGFR1 signaling in the presence of lactose, while mannose had virtually no effect on FGFR1 activation by galectins (Fig. S9). We performed signaling studies with recombinant gal-8_N-CRD_ and gal-8_C-CRD_ and neither gal-8_N-CRD_ nor gal-8_C-CRD_ induced FGFR1 nor ERK1/2 phosphorylation (Fig. 5B). We also confirmed that galectin-1, -3, -7, and -8 activate FGFR1 and receptor-downstream signaling in human U2OS-R1 cells (Fig. S10).

To investigate whether galectin-mediated receptor clustering provides a mechanism for FGFR1 activation, we engineered a set of synthetic galectins of different valency: monovalent and multivalent variants with artificially adjusted valency. We employed a streptavidin (SA)-based system for controlled protein oligomerization [32, 33]. Wild type SA is a tetramer capable of stable binding to four biotinylated ligands, ensuring protein tetramerization. Gal-8_N-CRD_ and gal-8_C-CRD_ were site-specifically biotinylated in the AviTag sequence using GST-BirA (Fig. S11) and assembled with recombinant SA, resulting in the tetravalent variants of galectin-8: gal-8_N-CRD_-SA and gal-8_C-CRD_-SA (Fig. 5C, D and F, G). Signaling studies revealed that while both monomeric gal-8_N-CRD_ and gal-8_C-CRD_ are largely deficient in FGFR1 activation, their tetravalent variants highly efficiently activate FGFR1 and receptor-downstream signaling (Fig. 5E and H). The surprising activation of FGFR1 signaling by gal-8_C-CRD_-SA, incapable of binding the receptor, is possibly due to an indirect effect caused by crosslinking of some FGFR1 binding partner by tetravalent gal-8_C-CRD_-SA.

Using the SA-based system, we also constructed engineered galectin-3 variants. Wild type galectin-3 forms pentamers or ligand-induced polymers using the N-terminal region [17]. We constructed an Avi-Tagged, monomeric galectin-3 variant lacking the native N-terminal oligomerization motif, gal-3_CRD_, and a tetravalent gal-3_CRD_-SA by self-assembling of a site-specifically biotinylated gal-3_CRD_ with SA (Fig. 5I and J, Fig. S12). Although gal-3_CRD_ retained the ability to induce FGFR1-dependent signaling, its activity was strongly enhanced by the gal-3_CRD_ tetramerization (Fig. 5K).

We studied whether the clustering-based mechanism of FGFR1 activation is also exploited by the prototypic galectin-1, which forms non-covalent dimers [17]. We prepared gal-1_CRD_, a monomeric galectin-1 variant composed exclusively of CRD and lacking the N-terminal sequence responsible for dimerization (Fig. 5L, Fig. S13A) [34]. We fused gal-1_CRD_ to a coiled coil (CC) sequence ensuring the self-assembly of gal-1_CRD_ into pentamers [35, 36], resulting in gal1_CRD_-CC.5x (Fig. 5L). Size exclusion chromatography confirmed that gal-1_CRD_ is monomeric, whereas gal-1_CRD_-CC.5x is pentameric (Fig. S13B). Signaling studies revealed that monomeric gal-1_CRD_ was completely inactive in triggering FGFR1 and ERK1/2 phosphorylation, but CC-mediated pentamerization restored its ability to activate FGFR1-dependent signaling (Fig. 5M). Unfortunately, we were unable to obtain galectin-7 variants with altered valency. The presence of lactose inhibited FGFR1 activation by all engineered galectins tested, while mannose had virtually no effect on galectin/FGFR1 signaling (Fig. S14).

All these data implicate that FGFR1 clustering promoted by multivalency of galectins is essential for FGFR1 activation and initiation of downstream signaling cascades.

### Galectins shape FGFR-dependent cell fate

**Fig. 6:**
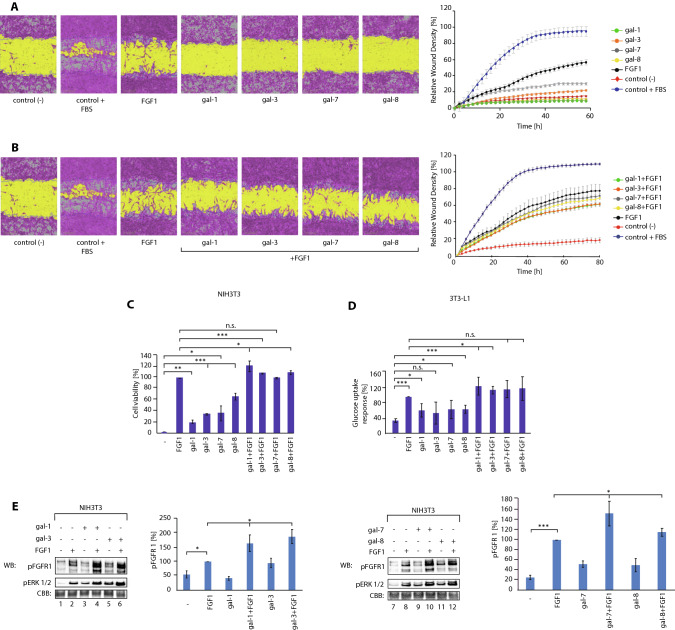
Galectins modulate cellular processes governed by FGFR1. **A** Wound-healing assays with NIH3T3 cells untreated [control (−)] or treated with serum [control (+)], FGF1 (100 ng/mL) or galectins (20 μg/mL) for 24 h. Wound images were automatically acquired every 2 h. Data were analyzed for spatial cell density in the wound area using the IncuCyte software package. At least three independent experiments were quantified. **B** Effects of galectins (5 μg/mL) on FGF1-induced cells migration (100 ng/mL). **C** Effect of FGF1 (20 ng/mL), galectins (20 μg/mL) and a mixture of FGF1 (20 ng/mL)/galectin (20 μg/mL) on NIH3T3 cell viability assessed with the Presto Blue Cell Viability Reagent. Mean values ± SEM from at least three independent experiments are shown. Statistical analyses were performed with Student’s *t* test (*p < 0.05; **p < 0.005 and ***p < 0.001). **D** Effects of FGF1 (20 ng/mL), galectins (50 μg/mL) and a mixture of FGF1 (20 ng/mL)/galectin (50 μg/mL) on glucose uptake by adipocytes. Mean values ± SEM from at least three independent experiments are shown. Statistical analyses were performed with Student’s *t* test (*p < 0.05; **p < 0.005 and ***p < 0.001). **E** Effects of galectins on FGFR1 signaling at saturating concentrations of FGF1 (100 ng/mL). Serum-starved NIH3T3 cells were treated with FGF1, recombinant galectins (20 μg/mL), or mixtures of these proteins for 15 min. Cells were lysed and analyzed with WB using the indicated antibodies. CBB served as a loading control. Results were quantified, normalized to FGF1 and mean pFGFR1 level from at least three independent experiments ± SEM are shown. Statistical analyses were performed with Student’s *t* test (*p < 0.05; **p < 0.005 and ***p < 0.001)

We next determined the cellular consequences of galectin-induced changes in FGF/FGFR signaling. Using a “scratch assay”, we found that, despite their high capacity to activate FGFR1 and receptor-downstream signaling pathways, none of the galectins tested significantly induced cell motility (Fig. 6A). To assess the effect of galectins on FGF1-mediated cell migration, we performed a “scratch assay” with FGF1 incubated together with galectins. We observed a slight inhibition of FGF1-induced cell migration by galectin-1 and -3 (Fig. 6B).

We measured the ability of the galectins alone to alter the number of viable cells and tested their effect on the number of viable cells after FGF1 treatment. As shown in Fig. 6C, all four galectins studied were able to elevate the number of viable cells, albeit to different extent, with galectin-8 showing the highest effect. Interestingly, the combination of galectin-1, -3, and -8 with FGF1 enhanced the number of viable cells more effectively than single proteins (Fig. 6C).

FGF1, by acting through FGFRs, is able to stimulate glucose uptake by adipocytes [37, 38]. We determined the metabolic activity of the galectins tested by measuring their effect on glucose uptake by model L3T3 adipocytes [37, 39, 40]. We observed a significant stimulation of glucose uptake by galectin-1, -7, and -8 (Fig. 6D). In addition, we detected enhanced glucose uptake by mixtures of FGF1 with galectin-1 and -3 compared to treatments with single proteins (Fig. 6D).

Since we observed synergistic effect of FGF1 and galectins on cell proliferation and glucose uptake, we wondered whether these effects might be due to increased activation of FGFR1 by the combinations of FGF1 and the studied galectins. To this end, we subjected serum-starved cells to receptor-saturating concentrations of FGF1 together with galectins and assessed the degree of FGFR activation with western blotting. We observed a significantly increased pFGFR1 signal in cells treated with FGF1/galectin mixtures compared to treatment with FGF1 alone (Fig. 6E). Interestingly, we observed that the pattern of pFGFR1 signal differed upon treatment with FGF1 and FGF1/galectin mixtures (Fig. 6E). The presence of mannose had no effect on FGF1/galectin signaling, while lactose restored the extent of pFGFR1 to the level observed for FGF1 alone, with a weaker inhibitory effect of lactose detected for galectin-8 (Fig. S15).

These data suggest that galectins modulate FGF/FGFR cellular processes through direct action on the receptor. Our data implicate that the cellular consequences of galectin-induced FGFR signaling largely differ from those achieved by the canonical ligand. While FGF1/FGFR signaling simultaneously triggers cell migration, division and glucose uptake, galectin/FGFR signaling stimulates cell division and glucose uptake without altering cell motility. Our data suggest that galectins are able to activate a pool of FGFRs that is inaccessible to stimulation by FGF1, resulting in an additive effect of FGF1 and galectins on some FGFR-dependent cellular processes.

## Discussion

The FGF/FGFR signaling units and galectins govern critical cellular processes and are ultimately implicated in human diseases, especially cancers [4, 14, 16, 17, 41–46]. For a long time, these diverse groups of proteins were treated separately. The first functional link between galectins and FGF signaling was reported by Markowska et al. [20], showing that FGF2-dependent angiogenesis is modulated by galectin-3. Subsequently, a role of galectin-3 in the modulation of FGF21 signaling through FGFR1 and Klotho-β was demonstrated [19]. Recently, we have provided the first evidence for a direct interplay between galectin-1 and -3, and FGFR1. We have shown that these galectins directly bind the N-linked sugar chains of FGFR1 activating it, modifying receptor endocytosis and altering cell proliferation and apoptosis [21]. Although these initial reports indicated the presence of a novel N-glycan/galectin regulatory module within FGFR signaling, a comprehensive analysis of the interaction between FGFRs and galectins has not been conducted to date.

**Fig. 7:**
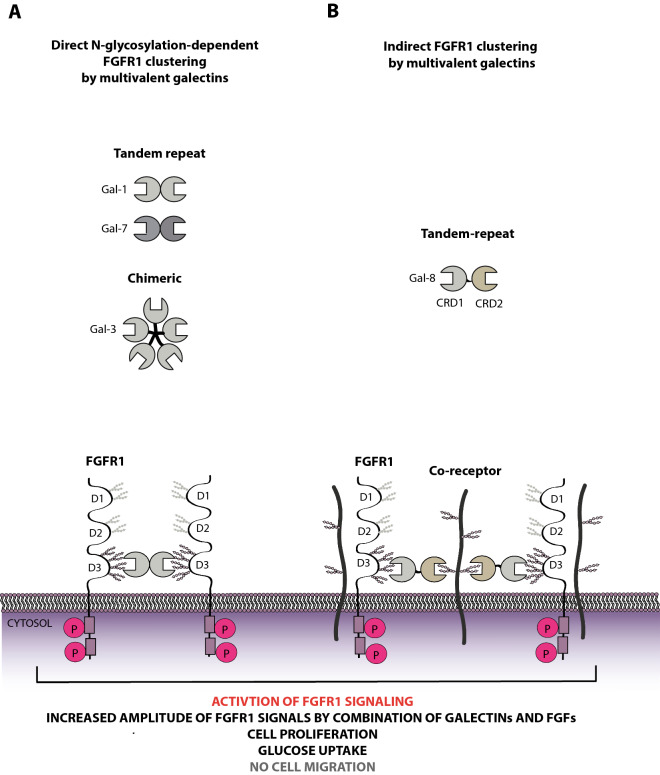
A hypothetical model of the clustering-mediated mechanism of FGFR activation and cellular signaling by galectins. All four identified galectins from all three human galectin families directly interact with the N-glycans of the D3 domain of FGFR1 causing receptor clustering and activation. **A** Single-specificity galectins from prototype and chimeric groups (galectins-1, -7 and -3) directly activate FGFR1 using a receptor cross-linking mechanism. Multivalency of galectins ensures FGFR1 clustering, which is achieved by the interaction of galectins with the N-glycans of the D3 domain of FGFR1 and results in FGFR1 activation and initiation of downstream signaling cascades. **B** Dual specificity tandem repeat galectin-8 recognizes FGFR1 with N-CRD and interacts with other oligomeric components of the plasma membrane (likely FGFR1 interactors) using C-CRD, which ensures indirect FGFR1 clustering and activation. Galectins are able to activate the FGFR1 pool not accessible for FGF1, intensifying FGFR1 signaling and enhancing cell division and glucose uptake, but at the same time having no effect on cell motility. As galectin-1, -3, -7, -8 largely differ in structure and valency, the architectures of FGFR1 clusters differ, which is reflected in the signaling potency of galectins and in the cellular effects triggered. Galectin/FGFR1 signaling effectively induces cell division and glucose uptake, but unlike FGF1/FGFR1 signaling is unable to trigger cell migration

Here we report for the first time that a specific set of human galectins from all three subfamilies (galectin-1, -3, -7, and -8) interact directly with N-linked sugar chains linked to FGF receptors, to regulate FGF/FGFR signaling and cell fate. Galectins bind with sub-micromolar affinities to the N-glycans of the membrane-proximal, FGF-recognizing, D3 domain of FGFR1, triggering differential clustering of FGFR1, activating the receptor and initiating downstream signaling cascades, as depicted on the scheme in Fig. 7. Interestingly, our data indicate that endogenous galectins might also partially block FGFR1 activation by FGF1 (Fig. 1A). Since cells simultaneously secrete several galectin family members, it is likely that a mixture of endogenous galectins might trap FGFR1 in clusters of heterogeneous architecture, in which activation of FGFR1 by FGF1 is less effective (for example due to reduced FGFR1 mobility or altered accessibility of FGF1 to the ligand-binding region of FGFR1). Recently, protein engineering approaches have been used to control the valency of galectins and thus the cellular effects triggered by these proteins [47, 48]. Based on these findings and on our experience in generating multivalent RTK ligands, we constructed monovalent and multivalent variants of the galectins studied [33, 35]. Using these proteins, we demonstrated that multivalency of galectins is strictly required for FGFR1 activation, indicating that N-glycosylation-dependent, direct FGFR1 clustering by multivalent galectins constitutes a novel mechanism of FGFR1 activation (Fig. 7). This mechanism appears predominant for single-specificity galectins (consisting of a single CRD differentially arranged in a multivalent format): chimeric galectin-3, prototype galectin-1, and likely galectin-7 (Fig. 7). Although receptor clustering by galectins and the ability of galectins to induce receptor activation have been previously reported for few RTKs, to our knowledge, our report provides the first experimental evidence that RTK clustering constitutes a mechanism for RTK activation by multivalent galectins [49–53].

Among galectins that interact with FGFR1, we identified a tandem repeat galectin-8 composed of two CRDs with distinct specificities, of which only gal-8_N-CRD_ directly binds N-glycans of FGFR1. While the wild type galectin-8 effectively activates FGFR1, monovalent gal-8_N_-_CRD_, although fully capable of FGFR1 binding, is unable to induce FGFR1 activation. Surprisingly, engineered tetravalent variants of both gal-8_N_-_CRD_ and gal-8_C-CRD_ activate FGFR1 and receptor-downstream signaling cascades, indicating a novel mechanism of FGFR1 stimulation by galectin-8. In this mechanism, galectin-8 binds FGFR1 with gal-8_N-CRD_ and some other, yet unknown cell surface co-receptor interacting with FGFR1 via gal-8_C-CRD_ to induce indirect clustering of FGFR1, leading to receptor activation (Fig. 7). FGFR1 interacts with a number of cell surface proteins, such as integrins, other RTKs or proteoglycans, which have been reported as galectin-binding partners and could therefore act as galectin-8 co-receptors for FGFR1 clustering [13, 54–57]. The involvement of co-receptors in RTK signaling mediated by galectins has been already suggested [58, 59]. The co-receptor employed by galectin-8 to activate FGFR1 is unknown and further studies should focus on its identification.

Transmission of signals by galectin/FGFR complexes has different consequences for the cell than canonical FGF/FGFR signaling. While FGF/FGFR induces a wide range of cellular responses: cell proliferation, cell migration and stimulates glucose uptake, the cellular effects of galectin/FGFR signaling appear narrower, with a predominant stimulation of glucose uptake and an increase in cell viability without affecting cell motility (Fig. 7). It should be noted that, due to the heterogeneity of N-glycans, the measured cellular effects of galectins may differ in part depending on cell origin, cell type, and physiological condition. Furthermore, we observed that partially distinct FGFR1 glycoforms are activated by FGF1 and galectins. Consistent with this, co-treatment of cells with saturating concentrations of FGF1 and galectins resulted in an additive effect, visible as increased level of activated FGFR1. These data indicate that the clustering mechanism employed by galectins enables activation of the FGFR1 pool, which is inaccessible to FGF1, resulting in a boosted mitogenic response of cells. These findings may be particularly relevant for the development of novel therapeutic strategies targeting simultaneously galectins and FGF/FGFR units to block uncontrolled division of cancer cells.

In summary, our data indicate that the N-glycans of FGFRs and FGFs constitute regulatory information that is differentially decoded by specific galectins to fine-tune cell physiology. The multivalency of galectins and their specificity for N-glycans appears to be a key factor in translating the information stored in the N-glycans of FGFRs and FGFs into specific cell activity. We demonstrate that receptor clustering provides a mechanism for FGFR activation by galectins. We also reveal a novel type of interaction between growth factors and galectins, which represents yet unknown regulatory mechanism in FGF/FGFR signaling. The multilayered action of multivalent galectins on FGFRs and FGFs fine-tunes cellular signaling and determines cell fate. Our findings might be relevant for the development of new therapeutic strategies, as galectins and FGF/FGFR are often upregulated in the same cancer types.

## Materials and methods

### Antibodies and reagents

The primary antibodies directed against FGFR1 (#9740), FGFR2 (#11835), FGFR3 (#4574), phospho-FGFR1 (pFGFR1; #3476), ERK1/2 (#9102), and phospho-ERK1/2 (pERK1/2; #9101) were from Cell Signaling (Danvers, MA, USA). The anti-tubulin primary antibody (#T6557) and the anti-GST antibody (#G1160) were from Sigma-Aldrich (St. Louis, MO, USA). Anti-human IgG (Fc) antibody coupled to HRP (#ab97225) was from Abcam (Cambridge, UK). The primary antibodies directed against FGFR4 (#sc-136988) and the anti-His-Tag primary antibodies (#sc-8036) were from Santa Cruz Biotechnology (Dallas, TX, USA). Secondary antibodies were obtained from Jackson Immuno-Research Laboratories (Cambridge, UK). Protein A Sepharose, Glutathione Sepharose, and Heparin Sepharose resins were from GE Healthcare (Chicago, IL, USA). Ni–NTA agarose and PrestoBlue™ Cell Viability Reagent (Thermo Fisher Scientific, Waltham, MA, USA). α-Lactose Separopore resin was from bioWORLD (Irving, TX, USA).

### Cells

Mouse embryo fibroblast cells (NIH3T3) were cultured in Dulbecco’s Modified Eagle’s Medium—DMEM (Thermo Fisher Scientific) supplemented with 10% fetal bovine serum (FBS) (Thermo Fisher Scientific) and antibiotics (100 U/mL penicillin, 100 μg/mL streptomycin). U2OS and USOS-R1-R4 cells were cultivated in DMEM (Biowest, Nuaille, France) supplemented with 10% FBS (Thermo Fisher Scientific) and antibiotics [100 U/mL penicillin, 100 μg/mL streptomycin, and geneticin (1 mg/mL)]. 3T3-L1 preadipocytes were maintained until 90% confluence. Next, the medium was exchanged to differentiation medium—DMEM (PAN-Biotech GmbH, Aidenbach, DE) supplemented with 10% FBS (Thermo Fisher Scientific), 0.5 mM isobutylmethylxanthine—IBMX (Sigma-Aldrich, Saint Louis, MO, USA), 1 μg/mL insulin, and 1 μM dexamethasone (Sigma-Aldrich), for 3 days. Next, adipocytes were maintained for maturation until day 12 in DMEM supplemented with 10% FBS and 1 μg/mL insulin. All cell lines were cultured in 5% CO_2_ atmosphere at 37 °C and were seeded onto tissue culture plates 1 day prior to the start of the experiments. JIMT-1, G292, and DMS114 cells were cultured as described previously [60, 61].

### Recombinant proteins

Genetic constructs allowing for expression of human galectins were prepared using the Gateway Cloning technique (according to the manufacturer’s protocol; Thermo Fisher Scientific), by recombination to pDEST17 or pDEST15 plasmids. His-tag and glutathione *S*-transferase (GST) fusions of galectins were expressed in *E. coli* BL21 CodonPlus(DE3)-RIL (Agilent Technologies, Santa Clara, CA, USA). Cells harboring the appropriate vectors were grown at 37 °C until OD_600_ = 0.8. Protein expression was induced by addition of 1 mM IPTG, and then cells were incubated at 16 °C (His-tagged galectins) or 25 °C (GST-tag galectins) overnight. Proteins were purified by affinity chromatography using Ni–NTA and Glutathione Sepharose resins, respectively.

For engineering of galectins valency, gal-8_N-CRD_ (Met^1^–Ser^152^), gal-8_C-CRD_ (Phe^187^–Trp^317^), and gal-3_CRD_ (Gly^108^–Leu^251^) were fused C-terminally with the AviTag and N-terminally with the HisTag. Proteins were expressed in *E. coli* BL21(DE3) pLysS strain (Agilent Technologies, Santa Clara, CA, USA). Cells were grown at 37 °C until OD_600_ = 0.8, then protein expression was induced by addition of 1 mM IPTG, followed by incubation of cells at 25 °C, overnight. Avi-tagged proteins were purified by affinity chromatography using Ni–NTA resin. The tetravalent SA and GST-BirA were purified and refolded as described in [33]. Site-specific biotinylation of Avi-tagged galectins, assembly of SA-based galectin tetramers and their subsequent purification was carried out as described recently by us [33]. The genetic construct for expression of gal-1_CRD_ (residues Ser^8^–Asp^135^ of the wild type galectin-1) was prepared using Phusion™ Site-Directed Mutagenesis Protocol. The protein was expressed in *E. coli* BL21 CodonPlus(DE3)-RIL (Agilent Technologies). Cells harboring the appropriate vector were grown at 37 °C until OD_600_ = 0.8. Expression was induced by addition of 1 mM IPTG and then cells were incubated at 30 °C for 4 h. Plasmid for expression of gal-1_CRD._CC.5x was prepared with Restriction-Free Cloning by the in-frame insertion of a sequence encoding pentamerizing coiled coil motif CC.5x to the gal-1_CRD_ expression vector [35, 36]. Cells were grown at 37 °C until OD_600_ = 0.3, followed by growth at 16 °C to OD_600_ = 0.8. Protein expression was induced by the addition of 1 mM IPTG, followed by incubation of cells at 16 °C overnight. Galectin-1 monomeric and multimeric variants were purified by affinity chromatography using Ni–NTA resin.

FGFR1-Fc-FGFR4-Fc and FGFR1 truncation lacking the D1 domain (FGFR1.D2.D3-Fc) were produced as described in [24]. Plasmids for construction of FGFR.D3-Fc (lacking the D1 and D2 domains) and FGFR1.GF-Fc (FGFR1-Fc mutant devoid of all N-glycosylation sites in the extracellular region) were prepared using restriction-free cloning and Phusion™Site-Directed Mutagenesis, respectively, using plasmid encoding FGFR1-Fc as a template. All FGFR-Fc variants were expressed in CHO cells and purified using Protein A Sepharose [24]. FGF1 and FGF2 were purified as reported in [35, 61].

The purity and the identity of all obtained proteins were confirmed by SDS-PAGE and western blotting. The oligomeric state of recombinant proteins was assessed by gel filtration [33, 35]. The detailed information about sequences of recombinant galectins used in this study can be found in the Supplementary Information.

### CBB staining

Polyacrylamide gels were stained for 1 h with CBB stain solution (1 g/l CBB R-250, 25% isopropanol, 10% acetic acid) and subsequently de-stained with Destainer solution (30% ethanol, 10% acetic acid).

### Pull-down

Purified His-Tagged galectins (10–50 μg) were bound to Ni–NTA or lactose–agarose resin in the Lysis Buffer (LB: 50 mM Tris, 150 mM NaCl, 1 mM EDTA, 0.1% Nonidet P-40, 1 mM PMSF, Protease Inhibitors Cocktail, pH 8.0). U2OS-R1, U2OS-R2, U2OS-R3, U2OS-R4, JIMT-1, DMS114, G292 cells were lysed in LB and clarified lysate was incubated with resin-bound proteins for 1 h at 4 °C with end over end rotation. Beads were washed with PBS, and bound proteins were eluted with SDS-PAGE sample buffer. Proteins were separated by SDS-PAGE and analyzed by western blotting.

Purified recombinant galectins bearing His-tag at the N-terminus (10 μg) were bound to lactose resin in PBS and incubated 1.5 h in cold room, end over end rotation. Resin was gently washed with PBS and bound proteins were eluted with SDS-PAGE sample buffer. Galectins were separated by SDS-PAGE and analyzed by western blotting.

### Galectin array with dot blot

Recombinant galectins (0.5 pmol), FGFRs, and Fc (0.2 pmol) were dot-blotted onto a PVDF membrane, previously activated with 70% EtOH. After blocking the membranes with 3% BSA, galectins arrays were incubated with FGFRs-Fc and the Fc (0.9 pM) overnight. Detection of galectin–FGFR-Fc complexes were performed with anti-Fc mAb-HRP for 1 h and chemiluminescence.

### Bio-layer interferometry (BLI)

To analyze the interaction between FGFRs and galectins, BLI measurements were conducted using Octet RED K2 system (ForteBio, San Jose, CA, USA). FGFRs-Fc and the Fc (25 μg/mL) were immobilized in PBS on Protein-A sensors in a pairwise manner (studied protein and the Fc on the reference sensor), and sensors were subsequently incubated with studied galectins (50 μg/mL). As a control, Fc was immobilized on Protein-A sensor and subsequently interaction with galectins (50 μg/mL) was analyzed. To assess the effect of sugars on the galectin/FGFRs interaction, BLI experiments were performed in the presence of 25 mM lactose or 25 mM mannose. For measurements of binding kinetics, sensor-immobilized proteins (25 μg/mL) were incubated with various concentrations of galectins (0.2–6 μM). The heterogeneous ligand (2:1) model was used for data fitting using Data Analysis 11 Software (Fortebio).

To study the impact of FGFR1 N-glycosylation on the interaction with galectins, PNGase F-deglycosylated receptor variants, glycosylation-deficient mutant of FGFR1 (25 μg/mL) was immobilized on Protein-A biosensors and incubated with studied galectins (50 μg/mL).

To locate binding sites for particular galectins on FGFR1, FGFR1-Fc (25 μg/mL) and FGFR1.D2-D3-Fc (25 μg/mL) were immobilized on Protein-A biosensors and incubated with galectins (50 μg/mL). Next FGFR1.D2-D3-Fc (25 μg/mL) and FGFR1.D3-Fc (25 μg/mL) were compered, as described above. In epitope binding approach, FGFR1.Fc (25 μg/mL) was immobilized on Protein-A biosensors and incubated with galectin-1, -3, -7, or -8 (50 μg/mL), whereas reference biosensor was incubated with the buffer only. Next, both biosensors were incubated with galectin-1 (50 μg/mL), galectin-3 (50 μg/mL), galectin-7 (50 μg/mL), or galectin-8 (50 μg/mL). To analyze the interaction between FGFR1.GF-Fc and FGF2, biotinylated FGF2 was immobilized on the high-precision Streptavidin biosensors (SAX), next the sensor was blocked with biocytin (0.04 mg/mL) and subsequently the interaction with FGFR1.GF-Fc (0.1 mg/mL) was measured.

### Dynamic light scattering (DLS)

Measurements were performed using a DynaPro NanoStar instrument (Wyatt Technology, CA) with FGFR1‐Fc (1 mg/mL), gal-1 (1 mg/mL), gal-3 (1 mg/mL), gal-7 (1 mg/mL), gal-8 (0.5 mg/mL), and mixtures of the proteins (mixtures of the proteins were incubated at RT for 10 min before measurements). A disposable microcuvette (Wyatt Technology) was used. Each measurement was performed at 20 °C in the PBS. DLS data were collected and analyzed using DYNAMICS V7 software (Wyatt Technology).

### FGFR activation and downstream signaling cascades

Serum-starved NIH3T3 cells were stimulated for 15 min with FGF1 (100 ng/mL) in the presence of heparin (10 U/mL) or various concentrations of recombinant galectins (1–20 μg/mL) or mixtures of studied proteins, at 37 °C. Cells were lysed in SDS-PAGE sample buffer, subjected to SDS-PAGE and visualized with western blotting using chemiluminescent substrate and ChemiDoc station (Bio-Rad). To study the impact of endogenous galectins on FGF/FGFR signaling, NIH3T3 cells were washed with 50 mM lactose or 50 mM mannose in DMEM for 15 min directly before incubation with FGF1. To assess the effect of sugars on the galectin-mediated activation of FGFRs, galectins were incubated with cells in the presence of 25 mM lactose or 25 mM mannose. Densitometric analysis of digital records was performed using the program ImageLab Software. At least three independent experiments were quantified.

### Cell viability

NIH3T3 was cultured in serum-free medium (DMEM) for 24 h. Cells were subsequently treated with galectin-1, -3, -7, -8 (5–20 μg/mL), FGF1 (20 ng/mL) with 10 U/mL heparin, or mix of studied proteins. Then, cells were incubated at 37 °C, 5% CO_2_ for 48 h, and cell viability was determined with Presto Blue Cell Viability Reagent (Thermo Fisher Scientific). At least three independent experiments were quantified.

### Glucose uptake

Differentiated 3T3-L1 cells seeded on the BioCoatTM poly-d-lysine 96-well (10,000 cells/well) (Corning, NY, USA) in DMEM without glucose (Thermo Fisher Scientific) and serum were stimulated with galectins (50 μg/mL), FGF1 (20 ng/mL), or mixtures of these proteins. The glucose uptake was determined with the Glucose Uptake-GloTM Assay (Promega, Madison, USA) according to the manufacturer’s protocol. At least three independent experiments were quantified.

### Wound healing assay

Cell migration was measured with the IncuCyte Scratch Wound Assay (Essen BioScience, Ann Arbor, MI, USA). Serum-starved NIH3T3 cells were seeded on a 96-well ImageLock plate and scratched with WoundMaker (Essen BioScience). Then, cells were stimulated with FGF1 (100 ng/mL, heparin 10 U/mL) and galectins (5 or 20 μg/mL) for 24 h. Every 2 h, images of the wounds were automatically acquired. The data were analyzed with respect to the spatial cell density in the wound area using the IncuCyte software package. At least three independent experiments were quantified.

### Statistics

Each of the experiments presented in the manuscript was repeated at least three times. Statistical analyses were performed with Student’s *t* test (*p < 0.05; **p < 0.005 and ***p < 0.001).

## Supplementary Information

Below is the link to the electronic supplementary material.

Supplementary data (Figs. S1–S15 together with Supplementary Figure Legends and Supplementary Materials and Methods). (DOCX 5747 KB)

## Data Availability

Data are available from the corresponding author upon reasonable request.

## Abbreviations

AKT: Protein kinase B
BLI: Biolayer interferometry
CBB: Coomassie brilliant blue
CC: Coiled coil
CRD: Carbohydrate recognition domain
DLS: Dynamic light scattering
DMEM: Dulbecco’s Modified Eagle’s Medium
ERK: Extracellular signal regulated kinase
FBS: Fetal bovine serum
Fc: Fragment crystallizable
FGFs: Fibroblast growth factors
FGFRs: Fibroblast growth factor receptors
GST: Glutathione *S*-transferase
HSPGs: Heparan sulfate proteoglycans
IBMX: Isobutylmethylxanthine
MEK: Mitogen-activated protein kinase/extracellular signal regulated kinase kinase
mTOR: Mammalian target of rapamycin
NIH3T3: Mouse embryo fibroblast cells
PI3K: Phosphoinositide 3-kinase
PLCγ: Phospholipase Cγ
SA: Streptavidin
SAX: Streptavidin biosensors
STAT: Signal transducer and activator of transcription
