# Supplementary material for: Receptor clustering by a precise set of extracellular galectins initiates FGFR signaling
Source: Cell Mol Life Sci. 2023 Apr 3;80(4):113. doi: 10.1007/s00018-023-04768-x (PMC10070233; doi:10.1007/s00018-023-04768-x)
Supplement: Supplementary file 1 — Supplementary data (Figs. S1–S15 together with Supplementary Figure Legends and Supplementary Materials and Methods). (DOCX 5747 KB) [file 18_2023_4768_MOESM1_ESM.docx]

**Supplementary Information for:**

**Receptor clustering by a precise set of extracellular galectins initiates FGFR signaling**

Dominika Zukowska^1^, Aleksandra Gedaj^1^, Natalia Porebska^1^, Marta Pozniak^1^, Mateusz Krzyscik^1^, Aleksandra Czyrek^2^, Daniel Krowarsch^2^, Malgorzata Zakrzewska^1^, Jacek Otlewski^1^ and Lukasz Opalinski^1^*

^1^Faculty of Biotechnology, Department of Protein Engineering, University of Wroclaw, Joliot-Curie 14a, 50-383 Wroclaw, Poland

^2^Faculty of Biotechnology, Department of Protein Biotechnology, University of Wroclaw, Joliot-Curie 14a, 50-383 Wroclaw, Poland

*Correspondence should be addressed to L.O ([lukasz.opalinski@uwr.edu.pl](mailto:lukasz.opalinski@uwr.edu.pl))

**Supplementary Figure Legends:**

**Figure S1. The effect of mannose on FGF1 signaling.** Serum-starved NIH3T3 cells were washed with 50 mM mannose prior the supplementation of cells with different concentrations of FGF1. Cells were lysed and analyzed with WB using indicated antibodies. CBB served as a loading control.

**Figure S2. Isolation of recombinant human galectins. A.** CBB stained of purified His-tagged or GST-tagged human galectins. **B**. WB analyses of isolated galectins using anti-His-Tag and ant-GST antibodies confirming identity of purified proteins. **C**. Lactose agarose pull down confirming activity of isolated galectins. Galectins were incubated with lactose agarose resin, resin was washed, bound proteins were eluted and analyzed with SDS-PAGE and CBB staining.

**Figure S3. The scheme of the galectin array experimental approach used for the identification of galectins interacting with FGFRs.** In the first step recombinant galectins were spot-immobilized onto PVDF membranes, which were then blocked to reduce non-specific binding of studied proteins. Next, galectin-spotted membranes were incubated with equimolar concentrations of the Fc fragment or FGFR-Fc. After washing the Fc or FGFRs-Fc bound to particular galectin were detected with anti-Fc antibody and chemiluminescence.

**Figure S4. Interaction between galectins and FGFR1 expressed by distinct human cell lines.** Galectin -1, -3, -7 and -8 (50 μg) were immobilized on lactose agarose resin and incubated with JIMT-1, DMS114 and G292 cell lysates. After extensive washing, resin-bound proteins were eluted and analyzed with SDS-PAGE and western blotting.

**Figure S5. BLI measurements of the interaction between FGFR1-Fc from HEK 293 cells and galectins.** FGFR1-Fc produced by HEK 293 cells and thus bearing human type of N-glycosylation was immobilized on the Protein-A BLI biosensors and incubated with galectin-1, -3, -7 and -8 (50 μg/ml). Recombinant Fc fragment was used for the reference biosensor.

**Figure S6. Isolation of N-glycosylation deficient mutant of FGFR1. A**. CBB-stained gel with Protein-A resin-purified FGFR1.GF-Fc, a receptor variant with all eight N-glycosylation sites mutated to alanines. **B**. BLI analysis of the interaction of SAX sensor-immobilized biotinylated FGF2 and FGFR1.GF-Fc, confirming functionality of prepared receptor variant.

**Figure S7. BLI measurements of the interaction between human FGFR1-Fc and galectins in the presence of lactose or mannose.** FGFR1-Fc was immobilized on the Protein-A BLI biosensors and incubated with galectin-1, -3, -7 and -8 (20 μg/ml) in the presence of lactose (25 mM) or mannose (25 mM).

**Figure S8. Preparation of FGFR1 truncations.** CBB stained gels of SDS-PAGE analyses of purified FGFR1 truncated variants: FGFR1ΔD1-Fc (**A**) and FGFR1ΔD1-D2-Fc (**B**) and their PNGase F mediated de-glycosylation.

**Figure S9. The effect of lactose and mannose on wild type galectins’ signaling.** Serum-starved NIH3T3 cells were incubated with galectins in the presence of lactose or mannose (25 mM). Cells were lysed and analyzed with WB using indicated antibodies. CBB served as a loading control.

**Figure S10**. **Effects of galectins on FGFR1 signaling in human U2OS-R1 cells**. Serum-starved U2OS-R1 cells were treated with FGF1 (100 ng/mL, control) or recombinant galectins. Cells were lysed and analyzed with WB using the indicated antibodies. CBB served as a loading control.

**Figure S11. Site-specific biotinylation of recombinant gal-8_N-CRD_ or gal-8_C-CRD_.** CBB-stained gels of GST-BirA biotinylated Avi-Tagged gal-8_N-CRD_ or gal-8_C-CRD._

**Figure S12. Site-specific biotinylation of gal-3_CRD._** CBB-stained gels of GST-BirA biotinylated AviTagged gal-3_CRD._

**Figure S13. Development of monovalent and multivalent galectin-1. A.** CBB-stained gels of recombinant monovalent gal-1_CRD_ and multivalent gal-1_CRD_.CC.5x **B**. Gel filtration analyses of the wild type galectin-1, the monovalent gal-1_CRD_ and the multivalent gal-1_CRD_.CC.5x**.** Experimental and theoretical MWs are shown.

**Figure S14**. **The effect of lactose and mannose on** **engineered galectins’ signaling.** Serum-starved NIH3T3 cells were incubated with engineered galectins in the presence of lactose or mannose (25 mM). Cells were lysed and analyzed with WB using the indicated antibodies. CBB served as a loading control.

**Figure S15. Effects of lactose and mannose on galectins/FGFR1 signaling.** Serum-starved NIH3T3 cells were treated with FGF1 (100 ng/mL), recombinant galectins (20 μg/mL), or mixtures of these proteins in the presence of 25 mM lactose or mannose for 15 min. Cells were lysed and analyzed with WB using the indicated antibodies. CBB served as a loading control.

**­**

**Supplementary Information: Supplementary Figures**

**
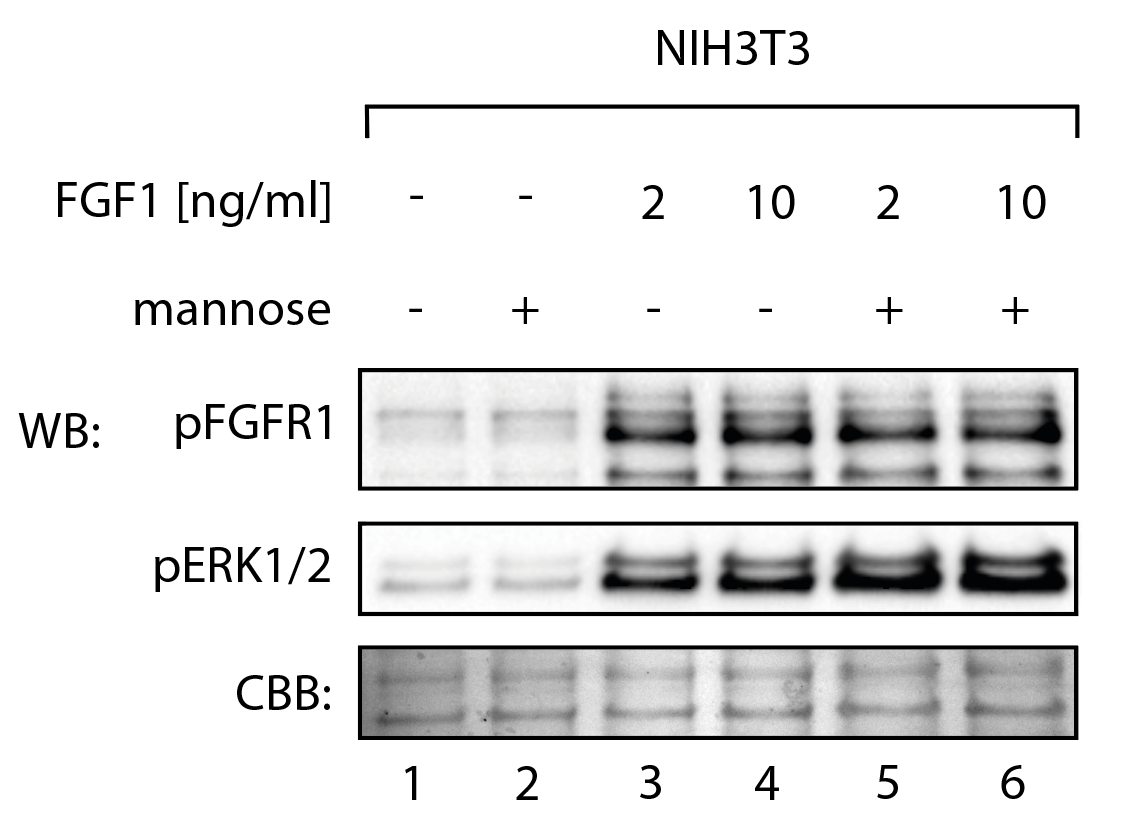
**

**Figure S1**


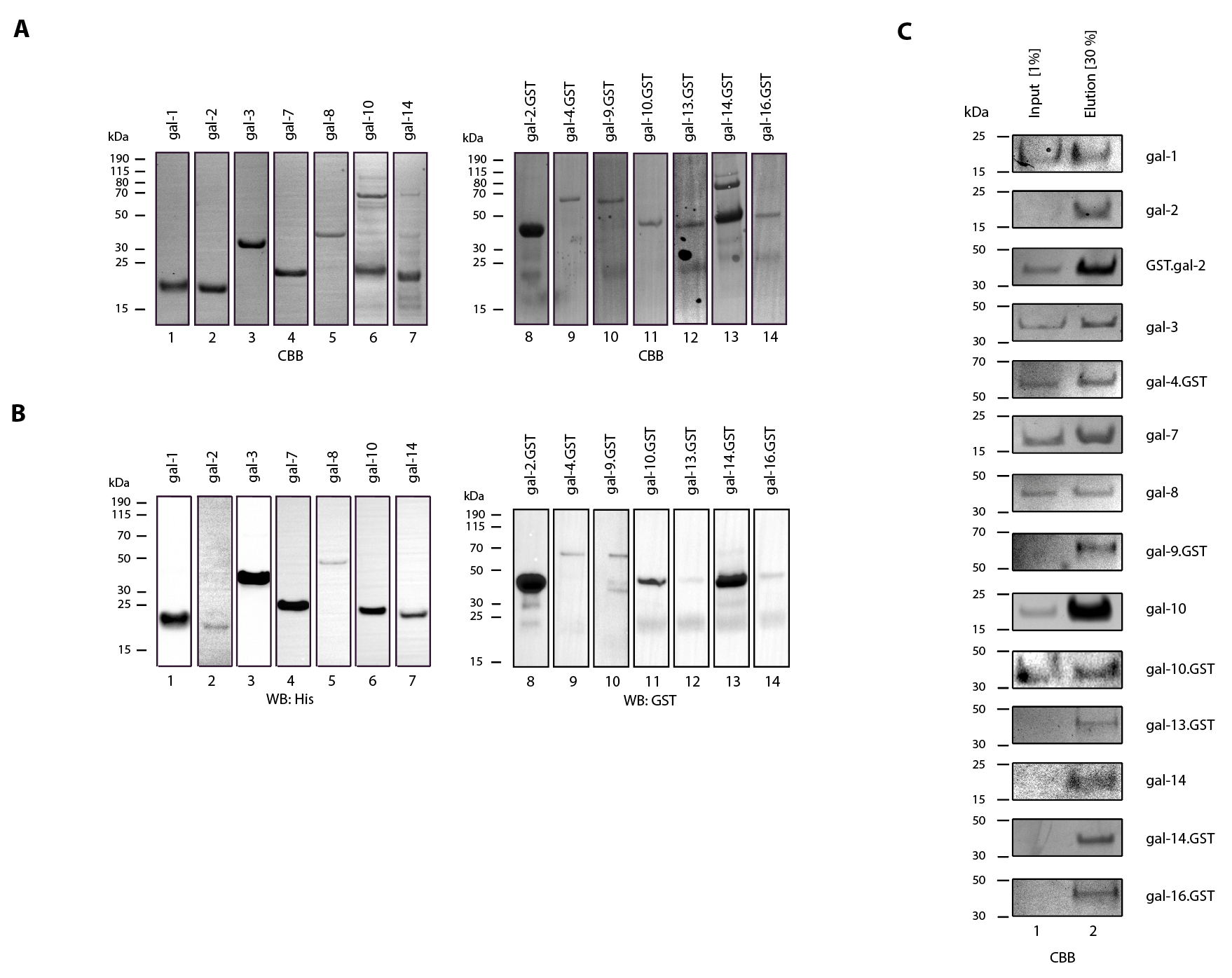


**Figure S2**

**
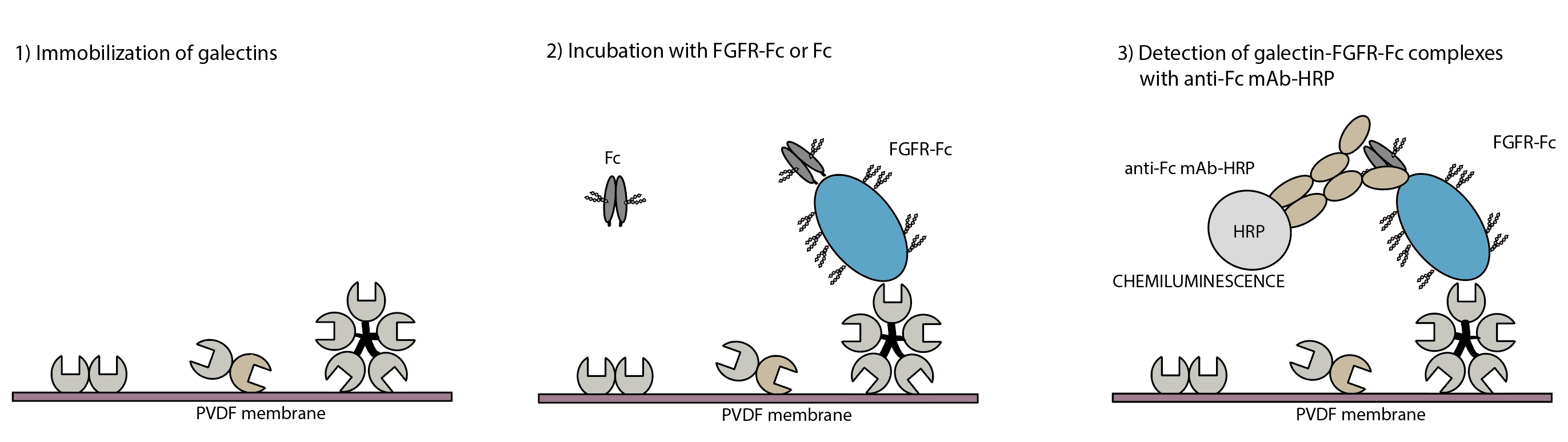
**

**Figure S3**

**
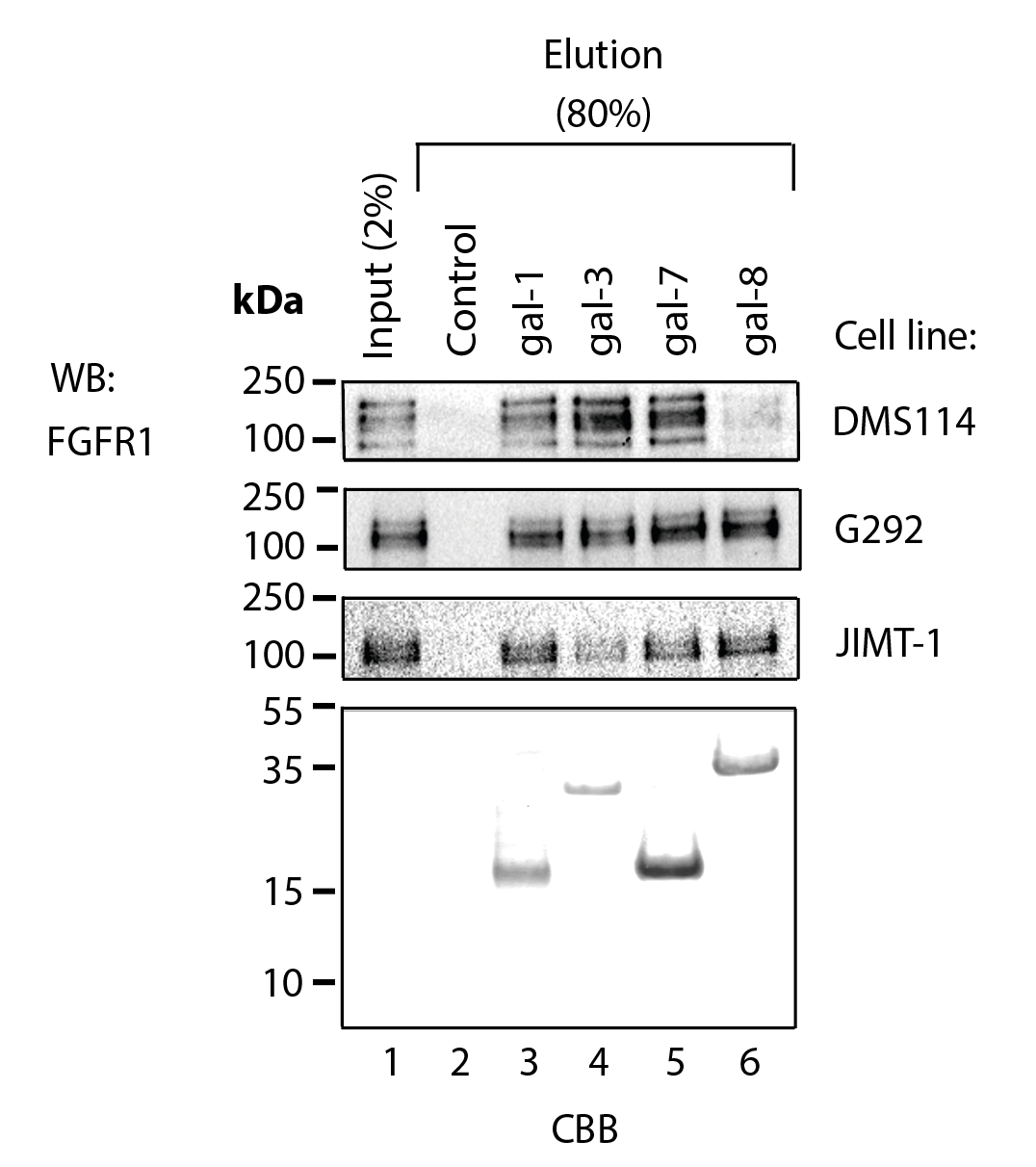
**

**Figure S4**

**
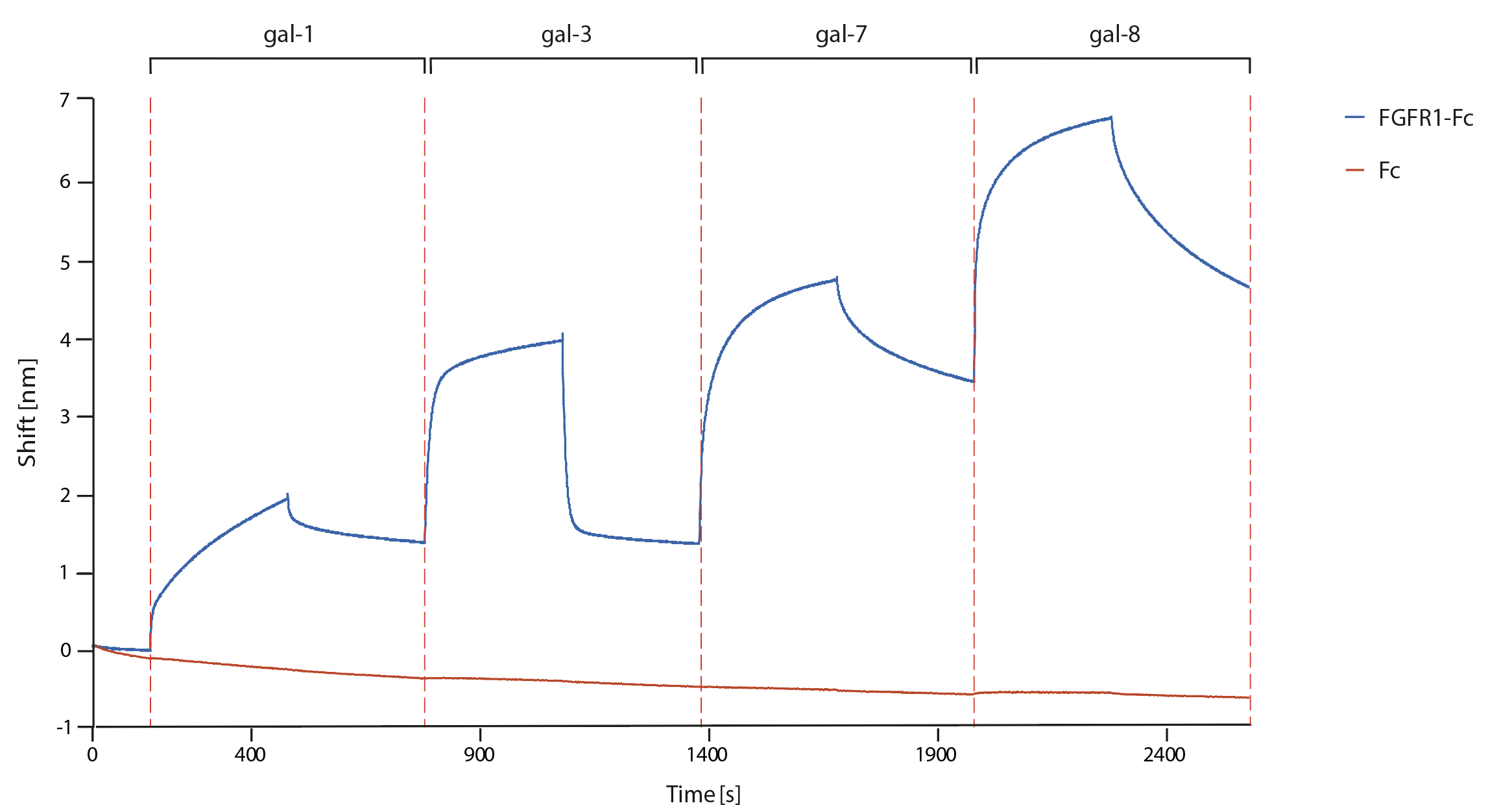
**

**Figure S5**

**
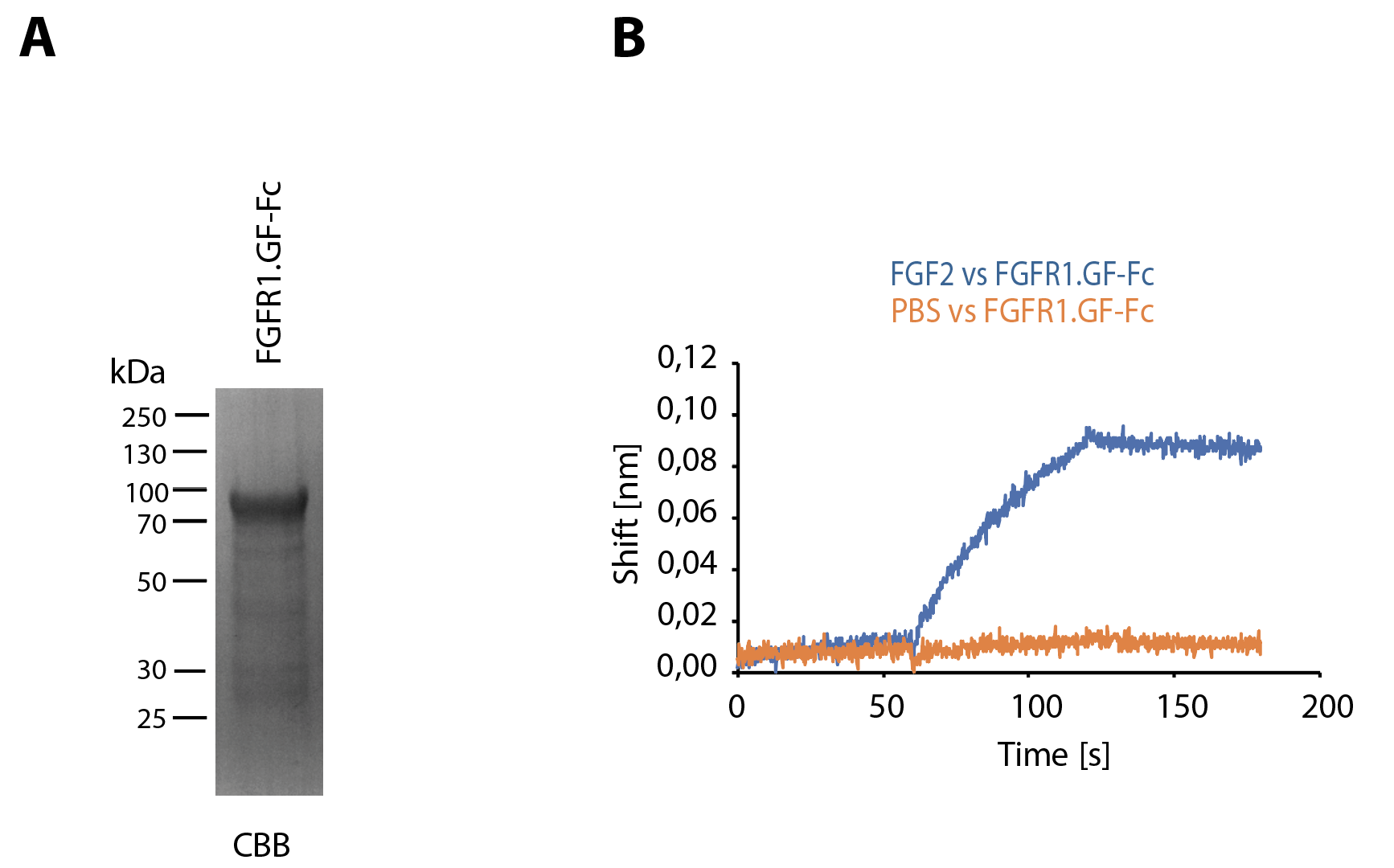
**

**Figure S6**

**
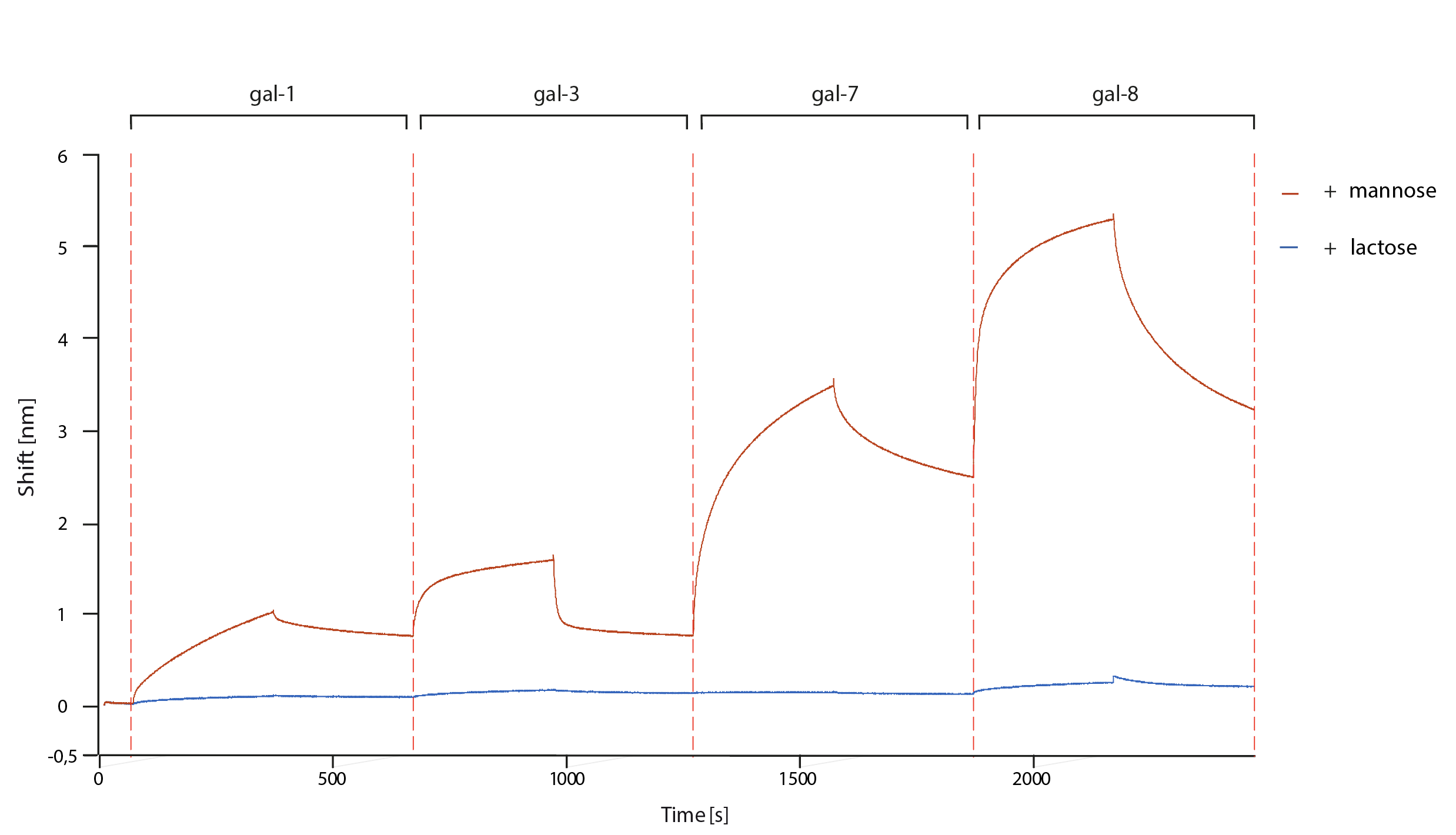
**

**Figure S7**

**
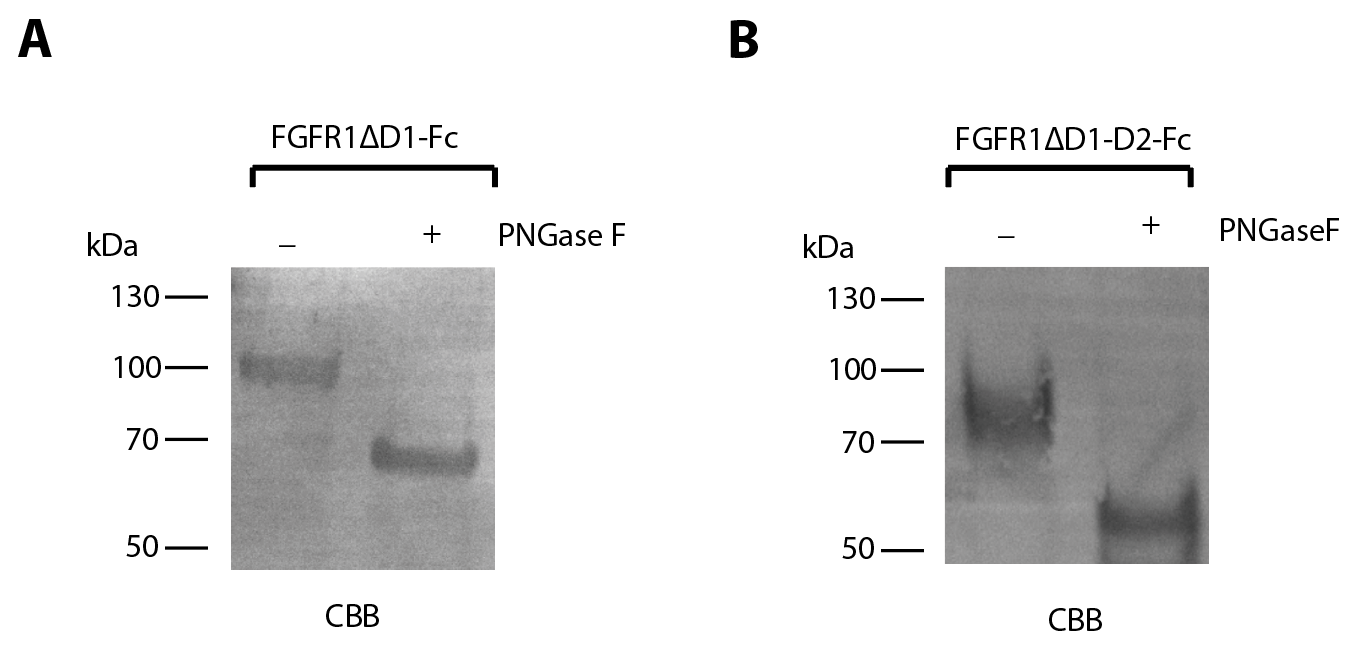
**

**Figure S8**

**
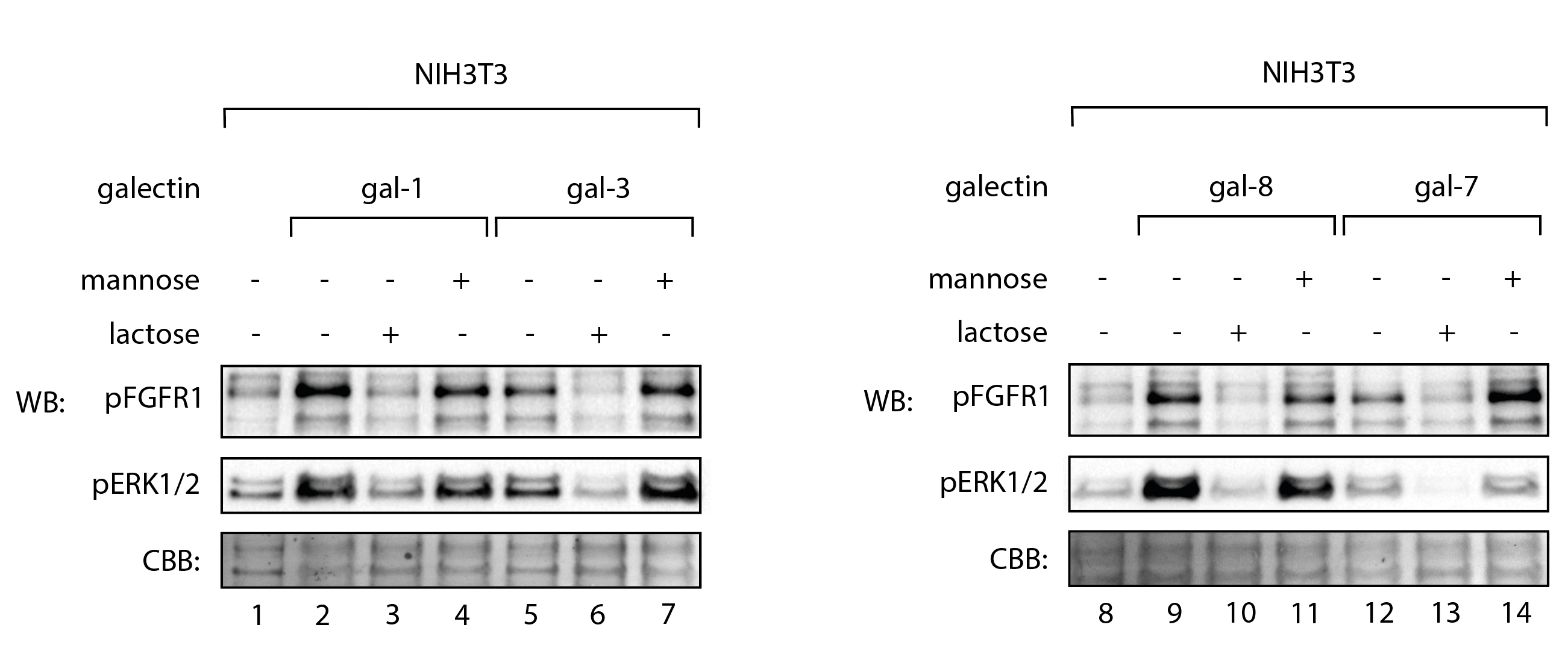
**

**Figure S9**

**
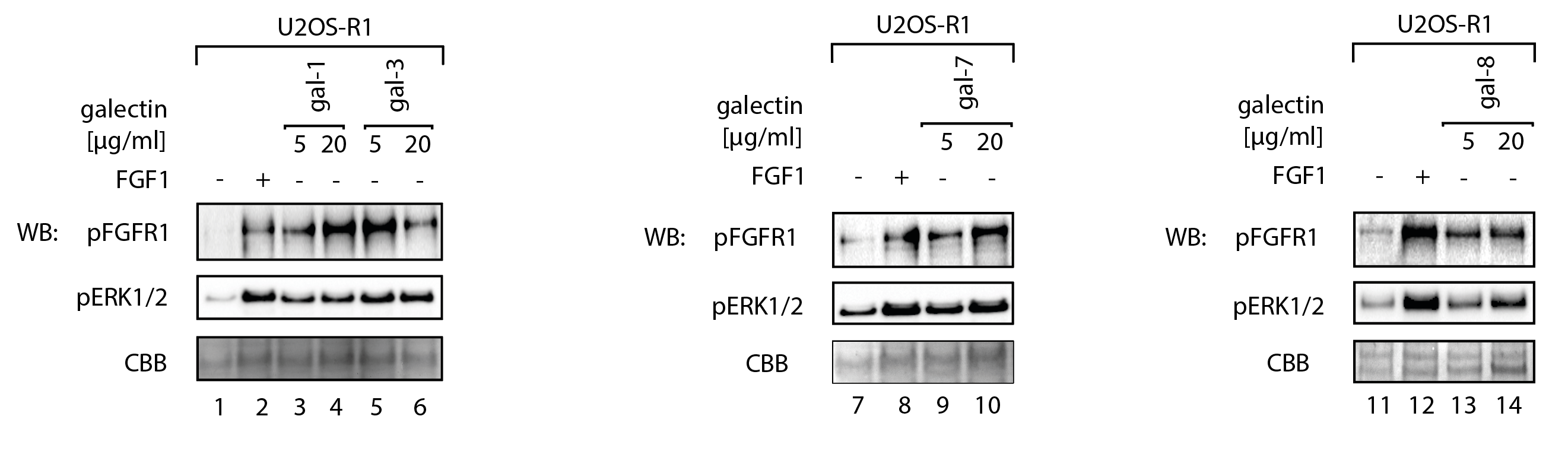
**

**Figure S10**

**
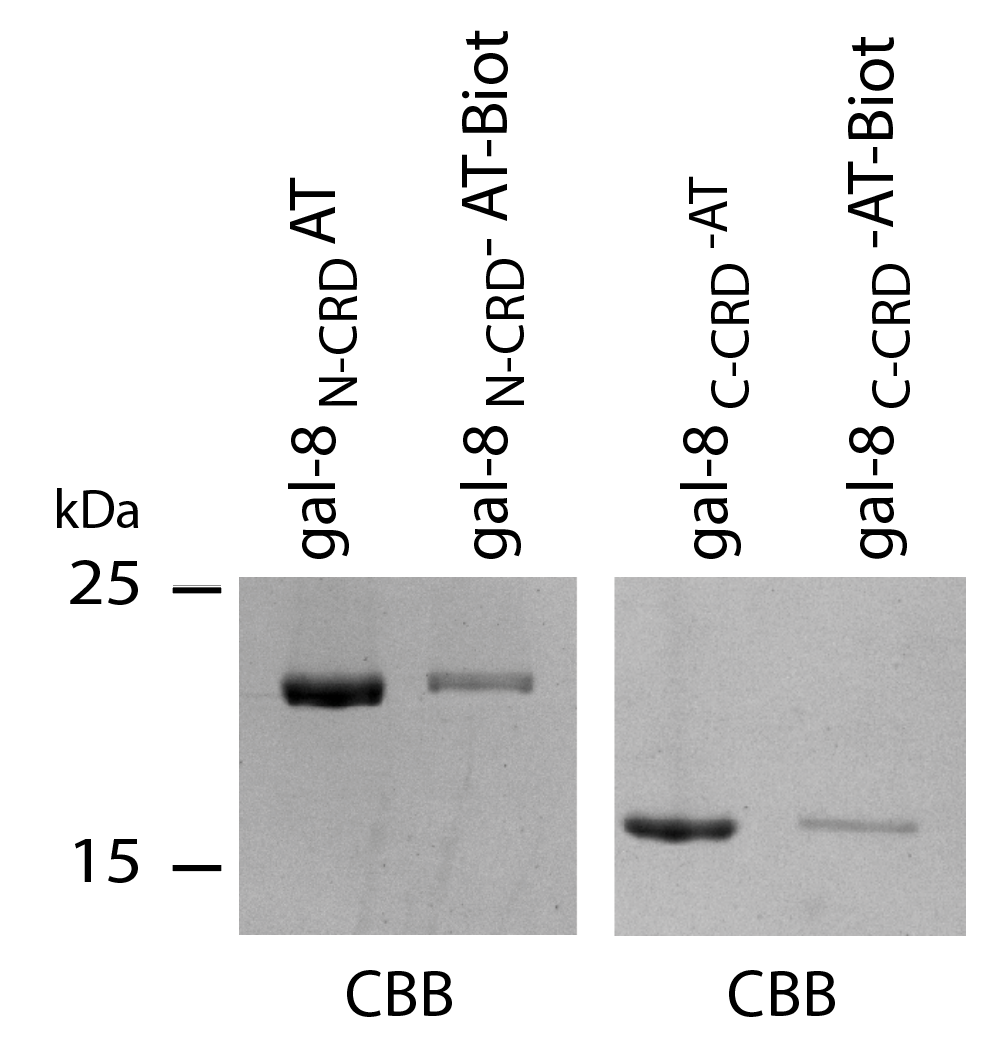
**

**Figure S11**

**
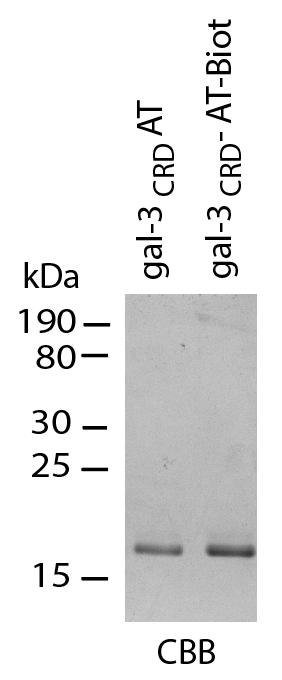
**

**Figure S12**


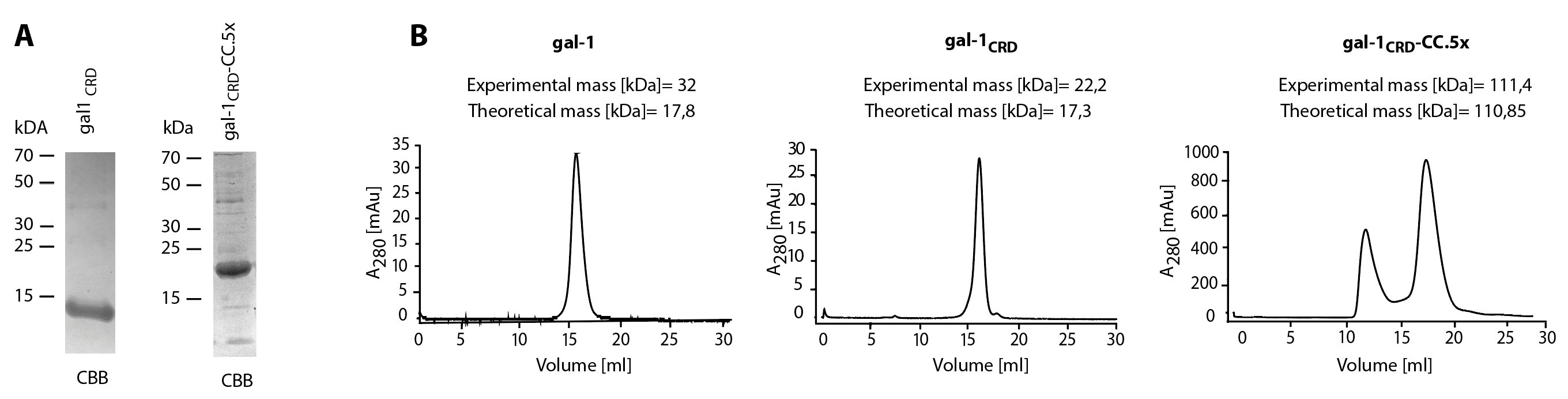


**Figure S13**

**
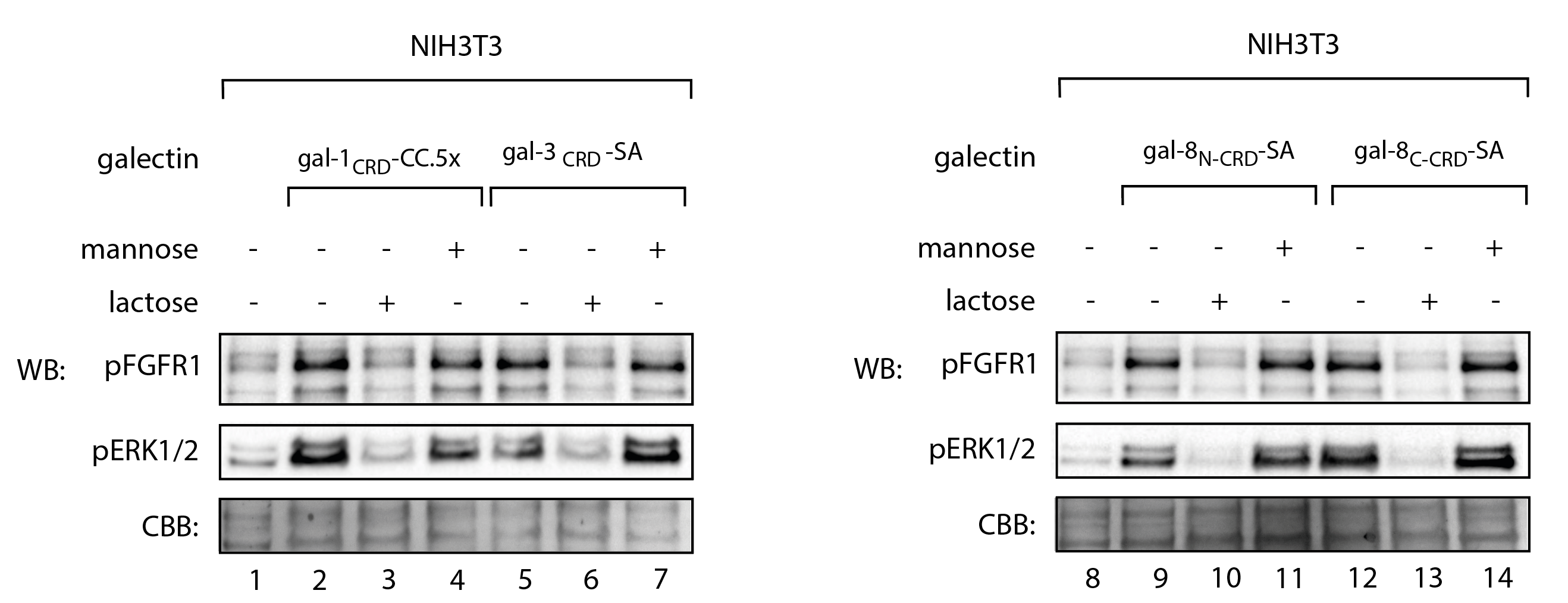
**

**Figure S14**

**
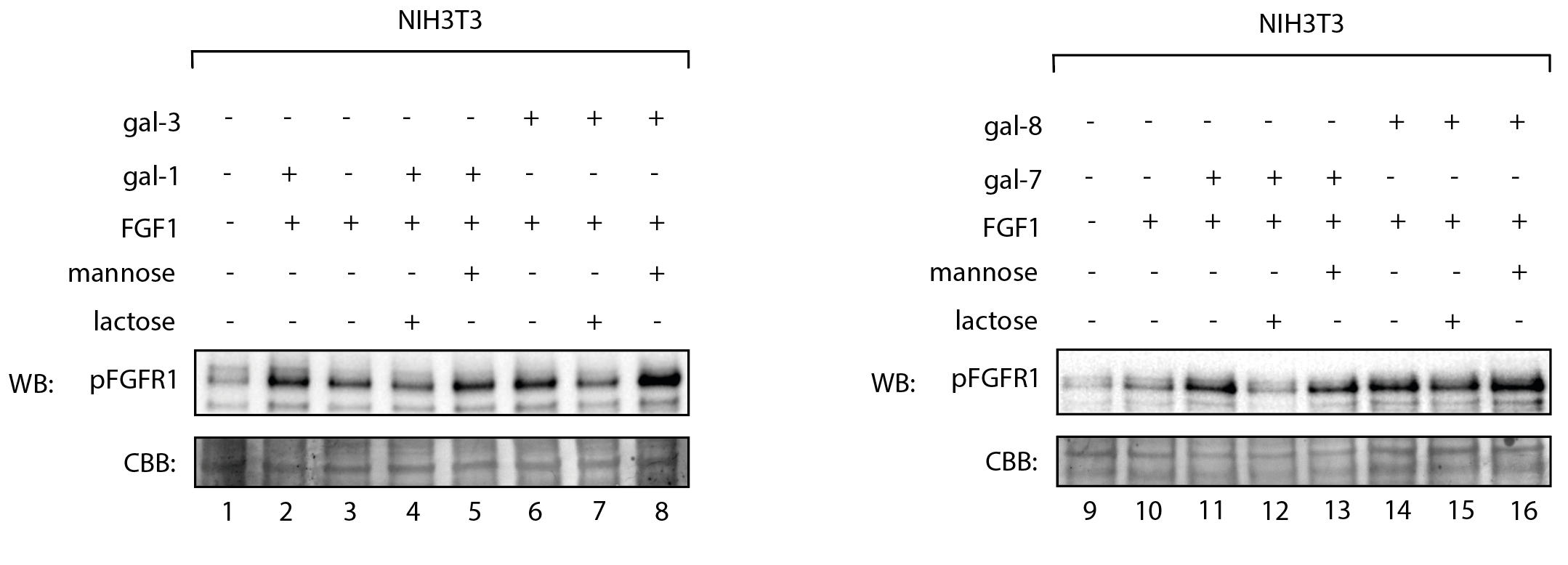
**

**Figure S15**

**Supplementary materials and methods**

**Table S1. Sequences of recombinant galectins used in this study.**

| **Protein name** | **Amino acid sequence** |
| --- | --- |
| Galectin-1 | MKHHHHHHPMSDYDIPTTENLYFQGAMACGLVASNLNLKPGECLRVRGEVAPDAKSFVLNLGKDSNNLCLHFNPRFNAHGDANTIVCNSKDGGAWGTEQREAVFPFQPGSVAEVCITFDQANLTVKLPDGYEFKFPNRLNLEAINYMAADGDFKIKCVAFD |
| Galectin-2 | MSYYHHHHHHLESTSLYKKAGSMTGELEVKNMDMKPGSTLKITGSIADGTDGFVINLGQGTDKLNLHFNPRFSESTIVCNSLDGSNWGQEQREDHLCFSPGSEVKFTVTFESDKFKVKLPDGHELTFPNRLGHSHLSYLSVRGGFNMSSFKLKE |
| Galectin-2.GST | MSPILGYWKIKGLVQPTRLLLEYLEEKYEEHLYERDEGDKWRNKKFELGLEFPNLPYYIDGDVKLTQSMAIIRYIADKHNMLGGCPKERAEISMLEGAVLDIRYGVSRIAYSKDFETLKVDFLSKLPEMLKMFEDRLCHKTYLNGDHVTHPDFMLYDALDVVLYMDPMCLDAFPKLVCFKKRIEAIPQIDKYLKSSKYIAWPLQGWQATFGGGDHPPKSDLVPRPWSNQTSLYKKAGSMTGELEVKNMDMKPGSTLKITGSIADGTDGFVINLGQGTDKLNLHFNPRFSESTIVCNSLDGSNWGQEQREDHLCFSPGSEVKFTVTFESDKFKVKLPDGHELTFPNRLGHSHLSYLSVRGGFNMSSFKLKE |
| Galectin-3 | MHHHHHHPMSDYDIPTTENLYFQGAMADNFSLHDALSGSGNPNPQGWPGAWGNQPAGAGGYPGASYPGAYPGQAPPGAYPGQAPPGAYPGAPGAYPGAPAPGVYPGPPSGPGAYPSSGQPSATGAYPATGPYGAPAGPLIVPYNLPLPGGVVPRMLITILGTVKPNANRIALDFQRGNDVAFHFNPRFNENNRRVIVCNTKLDNNWGREERQSVFPFESGKPFKIQVLVEPDHFKVAVNDAHLLQYNHRVKKLNEISKLGISGDIDLTSASYTMI |
| Galectin-4.GST | MSPILGYWKIKGLVQPTRLLLEYLEEKYEEHLYERDEGDKWRNKKFELGLEFPNLPYYIDGDVKLTQSMAIIRYIADKHNMLGGCPKERAEISMLEGAVLDIRYGVSRIAYSKDFETLKVDFLSKLPEMLKMFEDRLCHKTYLNGDHVTHPDFMLYDALDVVLYMDPMCLDAFPKLVCFKKRIEAIPQIDKYLKSSKYIAWPLQGWQATFGGGDHPPKSDLVPRPWSNQTSLYKKAGSMAYVPAPGYQPTYNPTLPYYQPIPGGLNVGMSVYIQGVASEHMKRFFVNFVVGQDPGSDVAFHFNPRFDGWDKVVFNTLQGGKWGSEERKRSMPFKKGAAFELVFIVLAEHYKVVVNGNPFYEYGHRLPLQMVTHLQVDGDLQLQSINFIGGQPLRPQGPPMMPPYPGPGHCHQQLNSLPTMEGPPTFNPPVPYFGRLQGGLTARRTIIIKGYVPPTGKSFAINFKVGSSGDIALHINPRMGNGTVVRNSLLNGSWGSEEKKITHNPFGPGQFFDLSIRCGLDRFKVYANGQHLFDFAHRLSAFQRVDTLEIQGDVTLSYVQIk |
| Galectin-7 | MSYYHHHHHHLESTSLYKKAGSMSNVPHKSSLPEGIRPGTVLRIRGLVPPNASRFHVNLLCGEEQGSDAALHFNPRLDTSEVVFNSKEQGSWGREERGPGVPFQRGQPFEVLIIASDDGFKAVVGDAQYHHFRHRLPLARVRLVEVGGDVQLDSVRIF |
| Galectin-8 | MSYYHHHHHHLESTSLYKKAGSMMLSLNNLQNIIYNPVIPFVGTIPDQLDPGTLIVIRGHVPSDADRFQVDLQNGSSMKPRADVAFHFNPRFKRAGCIVCNTLINEKWGREEITYDTPFKREKSFEIVIMVLKDKFQVAVNGKHTLLYGHRIGPEKIDTLGIYGKVNIHSIGFSFSSDLQSTQASSLELTEISRENVPKSGTPQLRLPFAARLNTPMGPGRTVVVKGEVNANAKSFNVDLLAGKSKDIALHLNPRLNIKAFVRNSFLQESWGEEERNITSFPFSPGMYFEMIIYCDVREFKVAVNGVHSLEYKHRFKELSSIDTLEINGDIHLLEVRSW |
| Galectin-9.GST | MSPILGYWKIKGLVQPTRLLLEYLEEKYEEHLYERDEGDKWRNKKFELGLEFPNLPYYIDGDVKLTQSMAIIRYIADKHNMLGGCPKERAEISMLEGAVLDIRYGVSRIAYSKDFETLKVDFLSKLPEMLKMFEDRLCHKTYLNGDHVTHPDFMLYDALDVVLYMDPMCLDAFPKLVCFKKRIEAIPQIDKYLKSSKYIAWPLQGWQATFGGGDHPPKSDLVPRPWSNQTSLYKKAGSMAFSGSQAPYLSPAVPFSGTIQGGLQDGLQITVNGTVLSSSGTRFAVNFQTGFSGNDIAFHFNPRFEDGGYVVCNTRQNGSWGPEERKTHMPFQKGMPFDLCFLVQSSDFKVMVNGILFVQYFHRVPFHRVDTISVNGSVQLSYISFQNPRTVPVQPAFSTVPFSQPVCFPPRPRGRRQKPPGVWPANPAPITQTVIHTVQSAPGQMFSTPAIPPMMYPHPAYPMPFITTILGGLYPSKSILLSGTVLPSAQRFHINLCSGNHIAFHLNPRFDENAVVRNTQIDNSWGSEERSLPRKMPFVRGQSFSVWILCEAHCLKVAVDGQHLFEYYHRLRNLPTINRLEVGGDIQLTHVQT |
| Galectin-10 | MSYYHHHHHHLESTSLYKKAGSMSLLPVPYTEAASLSTGSTVTIKGRPLACFLNEPYLQVDFHTEMKEESDIVFHFQVCFGRRVVMNSREYGAWKQQVESKNMPFQDGQEFELSISVLPDKYQVMVNGQSSYTFDHRIKPEAVKMVQVWRDISLTKFNVSYLKR |
| Galectin-10.GST | MSPILGYWKIKGLVQPTRLLLEYLEEKYEEHLYERDEGDKWRNKKFELGLEFPNLPYYIDGDVKLTQSMAIIRYIADKHNMLGGCPKERAEISMLEGAVLDIRYGVSRIAYSKDFETLKVDFLSKLPEMLKMFEDRLCHKTYLNGDHVTHPDFMLYDALDVVLYMDPMCLDAFPKLVCFKKRIEAIPQIDKYLKSSKYIAWPLQGWQATFGGGDHPPKSDLVPRPWSNQTSLYKKAGSMSLLPVPYTEAASLSTGSTVTIKGRPLACFLNEPYLQVDFHTEMKEESDIVFHFQVCFGRRVVMNSREYGAWKQQVESKNMPFQDGQEFELSISVLPDKYQVMVNGQSSYTFDHRIKPEAVKMVQVWRDISLTKFNVSYLKR |
| Galectin-13.GST | MSPILGYWKIKGLVQPTRLLLEYLEEKYEEHLYERDEGDKWRNKKFELGLEFPNLPYYIDGDVKLTQSMAIIRYIADKHNMLGGCPKERAEISMLEGAVLDIRYGVSRIAYSKDFETLKVDFLSKLPEMLKMFEDRLCHKTYLNGDHVTHPDFMLYDALDVVLYMDPMCLDAFPKLVCFKKRIEAIPQIDKYLKSSKYIAWPLQGWQATFGGGDHPPKSDLVPRPWSNQTSLYKKAGSMSSLPVPYKLPVSLSVGSCVIIKGTPIHSFINDPQLQVDFYTDMDEDSDIAFRFRVHFGNHVVMNRREFGIWMLEETTDYVPFEDGKQFELCIYVHYNEYEIKVNGIRIYGFVHRIPPSFVKMVQVSRDISLTSVCVCN |
| Galectin-14 | MSYYHHHHHHLESTSLYKKAGSMSSLPVPYTLPVSLPVGSCVIITGTPILTFVKDPQLEVNFYTGMDEDSDIAFQFRLHFGHPAIMNSCVFGIWRYEEKCYYLPFEDGKPFELCIYVRHKEYKVMVNGQRIYNFAHRFPPASVKMLQVFRDISLTRVLISD |
| Galectin-14.GST | MSPILGYWKIKGLVQPTRLLLEYLEEKYEEHLYERDEGDKWRNKKFELGLEFPNLPYYIDGDVKLTQSMAIIRYIADKHNMLGGCPKERAEISMLEGAVLDIRYGVSRIAYSKDFETLKVDFLSKLPEMLKMFEDRLCHKTYLNGDHVTHPDFMLYDALDVVLYMDPMCLDAFPKLVCFKKRIEAIPQIDKYLKSSKYIAWPLQGWQATFGGGDHPPKSDLVPRPWSNQTSLYKKAGSMSSLPVPYTLPVSLPVGSCVIITGTPILTFVKDPQLEVNFYTGMDEDSDIAFQFRLHFGHPAIMNSCVFGIWRYEEKCYYLPFEDGKPFELCIYVRHKEYKVMVNGQRIYNFAHRFPPASVKMLQVFRDISLTRVLISD |
| Galectin-16 | MSYYHHHHHHLESTSLYKKAGSMSFLTVPYKLPVSLSVGSCVIIKGTLIDSSINEPQLQVDFYTEMNEDSEIAFHLRVHLGRRVVMNSREFGIWMLEENLHYVPFEDGKPFDLRIYVCHNEYEVKVNGEYIYAFVHRIPPSYVKMIQVWRDVSLDSVLVNNGRR |
| Galectin-16.GST | MSPILGYWKIKGLVQPTRLLLEYLEEKYEEHLYERDEGDKWRNKKFELGLEFPNLPYYIDGDVKLTQSMAIIRYIADKHNMLGGCPKERAEISMLEGAVLDIRYGVSRIAYSKDFETLKVDFLSKLPEMLKMFEDRLCHKTYLNGDHVTHPDFMLYDALDVVLYMDPMCLDAFPKLVCFKKRIEAIPQIDKYLKSSKYIAWPLQGWQATFGGGDHPPKSDLVPRPWSNQTSLYKKAGSMSFLTVPYKLPVSLSVGSCVIIKGTLIDSSINEPQLQVDFYTEMNEDSEIAFHLRVHLGRRVVMNSREFGIWMLEENLHYVPFEDGKPFDLRIYVCHNEYEVKVNGEYIYAFVHRIPPSYVKMIQVWRDVSLDSVLVNNGRR |

***Gateway cloning of constructs allowing for production of recombinant galectins***

Genetic constructs allowing for expression of human galectins were prepared using the Gateway Cloning technique (according to the manufacturer's protocol; Thermo Fisher Scientific). In the first step in the PCR reaction DNA sequence encoding particular galectin was amplified with addition of *attB* flanking sites. Next step was BP reaction. *AttB*-flanked PCR product recombine with an *attP*-containing pDONR201 (kanamycin resistance) creating pENTRY vectors with *attL sites*. Reaction was performed with BP Clonase II Enzyme mix, ON, room temperature. Then, 0.5 µl of Protein K was added for 10 min at 37°C, in order to stop the reaction. The reaction mixture was transformed into *E. coli* DH10 competent bacteria cells. Then bacteria were plated on a LB-agar plate with the addition of proper antibiotic. Obtained single colonies were used to inoculate cultures for plasmid isolation. Then correct clones were used to perform an LR reaction between an *attL*-containing pENTRY clones and an *attR*-containing destination vector (pDEST15 for GST-fusion proteins or pDEST17 for His-tagged protein, both ampicillin resistance) to generate an expression clone. LR reaction was performed with LR Clonase II Enzyme mix, ON at room temperature followed by Proteinase K treatment and bacterial transformation in the same way as after BP reaction. The correctness of galectin coding sequences was confirmed with DNA sequencing.
